# Rupatadine-inhibited OTUD3 promotes DLBCL progression and immune evasion through deubiquitinating MYL12A and PD-L1

**DOI:** 10.1038/s41419-024-06941-x

**Published:** 2024-08-03

**Authors:** Ying Sui, Ziyang Shen, Xiaoyou Li, Ya Lu, SiTong Feng, Rong Ma, Jianzhong Wu, Changwen Jing, Zhuo Wang, Jifeng Feng, Haixia Cao

**Affiliations:** The Affiliated Cancer Hospital of Nanjing Medical University, Jiangsu Cancer Hospital and Jiangsu Institute of Cancer Research, Nanjing, China

**Keywords:** B-cell lymphoma, Apoptosis

## Abstract

The obstacle to effectively treating Diffuse Large B-cell Lymphoma (DLBCL) lies in the resistance observed toward standard therapies. Identifying therapeutic targets that prove effective for relapsed or refractory patients poses a significant challenge. OTUD3, a deubiquitinase enzyme, is overexpressed in DLBCL tissues. However, its role in DLBCL has not been investigated. Our study has brought to light the multifaceted impact of OTUD3 in DLBCL. Not only does it enhance cell survival through the deubiquitination of MYL12A, but it also induces CD8+ T cell exhaustion within the local environment by deubiquitinating PD-L1. Our findings indicate that the OTUD3 inhibitor, Rupatadine, exerts its influence through competitive binding with OTUD3. This operation diminishes the deubiquitination of both MYL12A and PD-L1 by OTUD3. This research unveils the central and oncogenic role of OTUD3 in DLBCL and highlights the potential clinical application value of the OTUD3 inhibitor, Rupatadine. These findings contribute valuable insights into addressing the challenges of resistant DLBCL cases and offer a promising avenue for further clinical exploration.

## Introduction

Diffuse large B-cell lymphoma, known as DLBCL, is the most prevalent lymphoma subtype, accounting for ~30–40 percent of all cases [1]. At present, survival rates for relapsed and refractory DLBCL patients who have undergone initial treatment are less than ideal, highlighting the necessity for novel therapeutic targets and approaches [2, 3]. Ubiquitination is a complex and critical process that regulates various cellular biological processes. During cancer progression, ubiquitination leads to the formation of signaling complexes, which is vital in the dynamic regulation of programmed cellular death. The prospects for understanding and developing ubiquitination-related targeted cancer therapies are promising [4, 5]. OTUD3 is a deubiquitinase enzyme that after being demonstrated by Lin Yuan et al in 2015 to be associated with PTEN stability and tumorigenesis, has since been shown to modulate tumor processes in various cancers [6–10]. Nevertheless, the function of OTUD3 in DLBCL has yet to be investigated or reported.

The tumor microenvironment (TME) holds significance for genesis and advancement of lymphomas [11, 12]. Examining the influence of T-cell immune checkpoints on DLBCL cell survival and the outcomes of immunotherapy holds substantial clinical significance [13]. The expression of PD-L1 has been substantiated to correlate with a less favorable prognosis in DLBCL patients [14]. Despite being influenced by factors such as individual patient variation and disease staging, clinical trials of PD-1/PD-L1 monotherapy or combination therapy in relapsed or refractory DLBCL patients have made substantial strides [15, 16].

In this study, we have undertaken the first systematic exploration of how OTUD3 regulates DLBCL cell survival through underlying mechanisms. Furthermore, we have revealed the intercellular regulation of CD8+ T cell exhaustion by OTUD3 in DLBCL cells and its potential mechanisms. Our investigation into OTUD3, along with its inhibitor rupatadine, has shown potential for offering innovative therapeutic choices in the field of targeted therapy and immunotherapy for patients with relapsed or refractory DLBCL [17].

## Materials and methods

### Patient specimen

Human DLBCL tissues and corresponding adjacent normal tissues were collected from the sample library of the Affiliated Cancer Hospital of Nanjing Medical University. The samples were frozen and stored in liquid nitrogen. The diagnosis of DLBCL was confirmed based on clinical manifestation and pathological examination. Informed consent was obtained from all subjects.

### Cell lines and cell culture

The lymphoma cell lines (Farage, OCI-LY-1 & Su-DHL-4) were provided by the Cell Repository of the Chinese Academy of Sciences located in Shanghai. The cells were cultivated in Roswell Park Memorial Institute medium (RPMI) 1640 (Gibco, USA), which was supplemented with 10 percent fetal bovine serum (FBS) (Ozfan, China, FBSKM0502) and GlutaMax. CD8+ T cells were grown in a medium enriched with IL-2 after activation with CD3/CD28 beads (Gibco, USA). These cells had been cultivated at 37 °C in a 5% carbon dioxide (CO_2_) incubation. Cells are treated with rupatadine (Targetmol, China) as needed, based on their respective IC50 values.

### CCK-8 assay

At the designated time, selected wells were augmented with CCK-8 reagent (APExBIO, Houston, USA). Following a 1-h incubation in the culture incubator, a microplate reader (ELx808, Bio Tek, USA) was utilized to assess absorbance values at 450 nm.

### Lactate dehydrogenase (LDH) assay

Steps followed the LDH assay kit instructions (Invitrogen, USA). Briefly, in the supernatant group, 20 μL of distilled water was added, while in the total LDH group, 20 μL of lysis buffer was added. After these procedures, the plate was incubated for 45 min. Following that, the supernatant (50 μL) was put in a new 96-well tray with an equal quantity of the reaction mixture. After 30 min, a stop solution was added, and absorbance values at 490 nm and 630 nm (background value) were recorded.

### EdU assay

The assessment of cell viability for all groups was conducted using the EdU assay kit (Beyotime, China). In summary, the EdU working solution was preheated and added to the samples. Afterward, each cell was stained with 4 percent paraformaldehyde. After fixation, the paraformaldehyde was removed, and each well was washed twice with cell stain buffer (Biolegend, USA). Subsequently, 1 mL of Phosphate Buffered Saline (PBS) (KeyGEN BioTECH, China) having 0.3% Triton X-100 (Beyotime, China) was used for cell permeabilization. Afterward, each well was washed with cell stain buffer for 1–2 cycles, and then the reaction solution was added. After incubation, the reaction solution was aspirated, and washing of cells was performed two times using cell stain buffer before analysis by flow cytometer (BD Bioscience, USA). FlowJo V10 was employed to determine the results.

### Annexin V and propidium iodide (PI) staining

DLBCL cell lines were washed and reconstituted in a Binding Buffer. Following that, each tube received FITC-Annexin V antibodies (Biolegend, USA) and Propidium Iodide (PI) Solutions (Biolegend, USA) with subsequent dark-incubation spanning 10 min. Then, each tube was filled with Binding Buffer and analyzed through a flow cytometer.

### Activated caspase-3/7 staining

DLBCL cell lines were washed and resuspended in cell stain buffer. Subsequently, each tube was added with Caspase-3/7 Green Reagent (Invitrogen, USA), and incubation was conducted at 37 °C in a light-protected atmosphere (with 5% CO_2_, 25 min). Afterward, SYTOX AADvanced (Invitrogen, USA) was added, and incubation lasted 5 min. Finally, the specimens were run using a flow cytometer.

### Mitochondrial membrane potential integrity assay

DLBCL cell lines were extracted and treated with 1 mL of cell stain buffer, and 1 μL of Image-iT TMRM Reagent (Invitrogen, USA) was added to each tube. Thereafter, incubation was conducted at 37 °C in a light-protected environment for 30 min. The samples were analyzed through a flow cytometer.

### Calcium Ion fluorescent probe assay

DLBCL cell lines were washed with HBSS (Hank’s Balanced Salt Solution) (KeyGEN BioTECH, China) solution twice. Fluo-4 AM (Yeasen, China) working fluid is added to these cells for a final concentration of 1 μM. Then, incubation was conducted for 30 min at 37 °C. After washing and resuspension in the HBSS solution, incubation was performed for another 30 min before analysis through a flow cytometer.

### Cell cycle analysis

DLBCL cell lines were washed and submerged in 75% ethanol overnight at −20 °C. Fixative extracted, a ready-to-use PI staining solution (KeyGEN BioTECH, China) containing RNase (Sangon Biotech, China) was used. After incubating the samples (37 °C, 30 min), the labeled cells passed through using a flow cytometer, and the data was processed with Modfit 5.

### Extraction of CD8+ T cells

Peripheral blood (8 mL) from healthy volunteers was drawn into an anticoagulant tube and then subjected to centrifugation at 1000 × *g* for 10 min. The lower layer of liquid, after dilution, was transferred into a clean centrifuge tube containing Ficoll separation solution (Solarbi, China). Subsequent centrifugation at 4 °C, 500 × *g* for 20 min, allowed for the aspiration of the mononuclear cell layer into a new tube. After washing the cells with PBS, erythrocyte lysis solution (Invitrogen, USA) was introduced in them, so they were dark incubated for 10 min. A final PBS wash was followed by resuspension of the cells in a complete culture medium.

The cell number was adjusted to 1 × 10^7^ in a total volume of 100 µL. Then, 10 µL of CD8+ T cell isolation magnetic beads (BioLegend, USA) were mixed and combined on ice for 15 min. After washing with a complete culture medium and collecting the precipitate by centrifugation, the collection material was placed on a magnetic stand (Invitrogen, USA), and it underwent two washes again.

### Activation of CD8+ T cells

After washing the CD3/CD28 beads (Gibco, USA), they were resuspended in 1 mL of complete culture medium, and IL-2 (PEPROTECH, USA) was added to achieve a final concentration of 10 ng/mL. The medium was then used to resuspend the extracted CD8+ T cells, and placed in a 37 °C, 5% CO_2_ incubator for further cultivation.

### Lymphoma cells and CD8+ T cells co-culture

DLBCL cells and the activated CD8+ T cells were adjusted to a cell number of 1 × 10^5^ each. The lymphoma cells were placed in a clean 24-well plate and then the suspension containing T cells was introduced. After 48 h incubation, T cells and DLBLC cells were separated with CD3+ T cell isolation magnetic beads (Invitrogen, USA) for flow cytometry staining.

### CD8, PD-1, and PD-L1 expression detection

After co-culturing with lymphoma cells, CD3+ T cell isolation beads served to obtain CD8+ T cells, which was followed by washing repeatedly with HBSS. Staining solutions for CD8 (CST, USA) and PD-1 (eBioscience, USA) fluorescent antibodies were prepared before incubation with cells in darkness for 15 min. The dye mixture was spun up and the cells were rinsed again with HBSS. Steps for PD-L1 staining were performed similarly. Finally, the cells were resuspended in HBSS and then analyzed using a flow cytometer.

### CD8+ T cells proliferation detection assay

After the co-culture and another process as described previously, the cells were resuspended in CFSE (Carboxyfluorescein succinimidyl ester) (Selleck, USA) working solution prepared based on the producer’s directions. These cells were then left in darkness for 20 min at the normal temperature. After this exposure, the staining solution was treated with a volume of culture media five times the size of the staining solution to remove unbound dye. The cells were then put back in a pre-warmed complete culture medium. A flow cytometer was then used to study the cells.

### Construction of stable OTUD3 knock-down and OTUD3 overexpressing lymphoma cell lines

The OTUD3 overexpression or knock-down lentivirus, purchased from Hanbio Tech (Shanghai, China), was used to transfect Farage, OCI-LY-1, and Su-DHL-4 cells. Subsequently, the cells remained continuously refined in a medium holding 2 μg/mL puromycin (Gibco, USA) to obtain stably transfected cell lines. The efficiency of OTUD3 knock-down or Overexpression was studied using Western blot (WB) analysis.

### Subcutaneous tumor model

Considering the effect size and standard deviation, the sample size for the animal study was determined following the recommendations of the Animal Ethics Committee in order to ensure the establishment of well-behaved animal models would possess adequate statistical power. BALB/c nude Mice were randomly divided into four groups (Vector/OE-OTUD3/Vector+Rupa/OE-OTUD3+Rupa, *n* = 5/5/4/4). The cells were rinsed 3 times with PBS. They were resuspended in a mixture of PBS and Matrigel (Absin, China, abs9490). The cells were then injected under the skin of 5-week-old BALB/c nude mice (GemPharmatech, Nanjing, China).

The C57BL/6 Eµ-myc transgenic mice were procured from The Jackson Laboratory (USA). Following the spontaneous onset of tumors, neoplasms were extracted and subjected to digestion, subsequently inoculated into a hexachrome plate. The cells were transduced with telomerase reverse transcriptase lentivirus for immortalization. C57BL/6 wild-type Mice were randomly divided into four groups (NC/NC+Rupa/NC + PD-L1/NC + PD-L1+Rupa, *n* = 4). Ultimately, these cells were subcutaneously injected into C57BL/6 wild-type murine hosts. All of the mice were mercifully murdered with CO_2_ at the designated time point, and the subcutaneous tumors were removed for size measurement. The cancer’s volume was approximated by a formula such as: (length × width^2^)/2.

### Lung metastasis model

The cells were cleaned repeatedly by PBS. They were revived in PBS before being injected into each mouse’s tail vein. BALB/c nude Mice were randomly divided into two groups (Vector+Rupa/OE-OTUD3+Rupa, *n* = 4). After 30 days, all the mice are euthanized using CO_2_, and their lungs are dissected to examine the extent of metastasis.

### Rupatadine treatment and anti-PD-L1 treatment in vivo

The treatment group received an oral gavage of rupatadine or Intraperitoneal administration of PD-L1 monoclonal antibody (BioXCell, the USA) 5 days a week for 2 weeks. The control group is orally gavaged or Intraperitoneal administrated with an equivalent volume of PBS.

### Co-immunoprecipitation assay and mass spectrometry

Flag magnetic beads (Millipore, USA) were collected and rinsed repeatedly using an IP lysis solution. Afterward, protein supernatant from IP lysis was added, and incubation was carried out overnight on a rotating shaker at 4 °C. The magnetic beads were extracted the next day by the magnet holder and cleansed three times with a washing solution. The following items are included in the assay kit (Thermo Scientific, USA), each wash lasted for 10 min, followed by a rinse with pure water. Finally, the proteins were eluted using an Elution Buffer Included within the assay kit for 3 min, and SDS (Sodium Dodecyl Sulfate) buffer (Beyotime, China) was poured and then heated at 95 °C in a metal bath for 5 min. Western blotting (WB) or mass spectrometry (MS) was performed to analyze the protein components. Protein identification services were provided by PTM BIO (Hangzhou, China).

### In vitro ubiquitination experiment

The prokaryotic expression plasmids GST-MYL12A, GST-PD-L1, and His-OTUD3 were transformed into BL21(DE3) cells. Positive clones were picked and shaken in cultures overnight. After further amplification, protein expression was induced with IPTG for 6 h. Subsequently, GST-MYL12A and His-OTUD3 proteins were purified using GST agarose beads and nickel columns. Subsequent experiments employed an in vitro ubiquitination modification kit (Ubiquitin-Proteasome Biotechnologies, the USA). Following a 2-h reaction at 37 °C, the reaction was terminated. After glutathione Sepharose was added, the mixture was rotated at 4 °C for 8 h and washed three times with a pull-down buffer. Finally, the SDS-PAGE sample buffer was added to the precipitate and heated in a water bath at 100 °C for 10 min.

### Rupatadine pulldown assay

Biotin-labeled rupatadine was utilized. In the in vitro pull-down assay, Farage cells were lysed in RIPA buffer containing protease inhibitors. Subsequently, labeled rupatadine was added and incubated at room temperature for 2 h at different concentrations. Excess pre-chilled methanol was then added, and the mixture was incubated at −80 °C for 30 min to precipitate proteins. After centrifugation at 14,000 × *g* for 15 min, the precipitated proteins were dissolved, and pull-down was performed using a biotin pull-down assay kit (Thermo Fisher, the USA). The beads were then washed, and the bound proteins were collected and detected through WB analysis.

As for the in situ pull-down experiment, Farage cells were seeded into a 6-well plate, after 12 h, rupatadine was added at various concentrations and further incubated for 6 h. Subsequently, cells were lysed in RIPA buffer containing protease inhibitors. Excess pre-chilled methanol was added after centrifugation, and the remaining steps were consistent with the in vitro pull-down procedure.

### Western blot analysis

After lysing cells with lysis buffer (Thermo Scientific, USA) containing protease and phosphatase inhibitors. For 20 min, the lysates were kept on ice. They were then spun up at 12,000 × *g* for 15 min at 4 °C, and the remaining residues were taken out to be measured with a spectrophotometer (NanoDrop One, Thermo Scientific, USA). Following that, an SDS sample buffer was inserted, and it was put over a metal bath at 95 °C for 10 min.

These were loaded into a precast gel (GenScript, China) and subjected to constant voltage electrophoresis at 140 V until the bromophenol blue reached its bottom. After that, proteins were moved into a PVDF sheet (Millipore, USA) using a wet transfer system at 300 mA for 2 h. Following this, the PVDF membrane was blocked with 5% BSA (Solarbio, China) for 30 min and then cut into appropriate sections for antibody incubation at 4 °C overnight. Afterward, the membrane was washed for 20 min in 1X PBST (New Cell & Molecular Biotech, China) on a shaker and subsequently incubated with the corresponding secondary antibodies. After another 30-min wash with 1X PBST on a shaker, the membrane was developed using an ECL substrate (New Cell & Molecular Biotech, China) and subjected to detection using a Chemiluminescence imaging system (Universal Hood II, BIO-RAD, USA). Please refer to the supplementary materials for the list of antibodies.

### RNA extraction and qRT-PCR

RNA Extraction was performed with an RNA extraction kit (Sangon Biotech, China) according to the manufacturer’s instructions. RNA content was determined using a spectrophotometer, followed by reverse transcription (Takara, Japan). Subsequently, the diluted product was mixed with SYBR Green pre-mix (Applied Biosystems, USA) based on the brand’s standards, and qRT-PCR was employed using a Fluorescence quantitative PCR instrument (LightCycler 480 Instrument II Roche, Switzerland).

### Statistical analysis and data visualization

The data were presented as the mean ± standard deviation. The experiments were replicated three or more times. The programming system R was used for data analysis (version = 4.2.3) with the ggpubr (version = 0.6.0) package. T-tests, one-way ANOVA, and to compare variations among multiple groups, a two-sided ANOVA was applied. When a value of *p* < 0.05 was chosen, the significance level was assessed. The R packages were utilized to present the information, ggplot2 (version = 3.4.3) and ggpubr, and data preprocessing was done using the dplyr (version = 1.1.2) and tidyverse (version = 2.0.0) packages.

### Molecular docking and visualization

The virtual docking of OTUD3 and rupatadine was carried out using AutoDock Tools (version 1.5.7). The PDB file for OTUD3 was achieved by using a protein databank (PDB), accession number: 4BOU, while the mol2 format for rupatadine was downloaded from DrugBank (accession number: DB11614) and converted to pdbqt format using Open Babel (version 3.1.1). The docking process followed the instructions provided on the author’s website, a binding energy below −5 kJ/mol is considered meaningful. The docking results were visualized using the online PLIP web tool.

### Immunohistochemistry (IHC) on tissue microarrays and data analysis

The DLBCL patient tissue microarrays were purchased from Servicebio (Wuhan, China). In brief, upon decomposition and dehydration of the tissue slides, the extraction of antigen was carried out, and treatment with 3% hydrogen peroxide. Subsequently, IHC was performed by outlining the sections with an immunohistochemistry pen, serum blocking, and overnight incubating with the primary antibody at 4 °C. The second antibody was incubated at ambient temperature the following day, followed by color development, counterstaining with hematoxylin, dehydration, clearing, and slide sealing for analysis.

Tissue microarray images were captured using a digital scanner for immunohistochemistry slides. The tissue measuring areas were automatically read using the Servicebio imaging tool. Positive staining was graded (i) as follows: no staining scored 0; weak positive staining scored 1; moderate positive staining scored 2; strong positive staining scored 3. Subsequently, the areas of weak, moderate, and strong positive staining, the total measured tissue area, and integrated optical density (IOD) values of positive staining, and positive staining area were calculated separately. The H-score is calculated as follows: H-SCORE = ∑ (pi × i) = (weak intensity area % × 1) + (moderate intensity area % × 2) + (strong intensity area % × 3), where ‘pi’ is the percent of a positive signal unit area and ‘i’ is the positive grading.

## Results

### OTUD3 is an oncogene in DLBCL and promotes the proliferation of diffuse large B-cell lymphoma cells

**Fig. 1:**
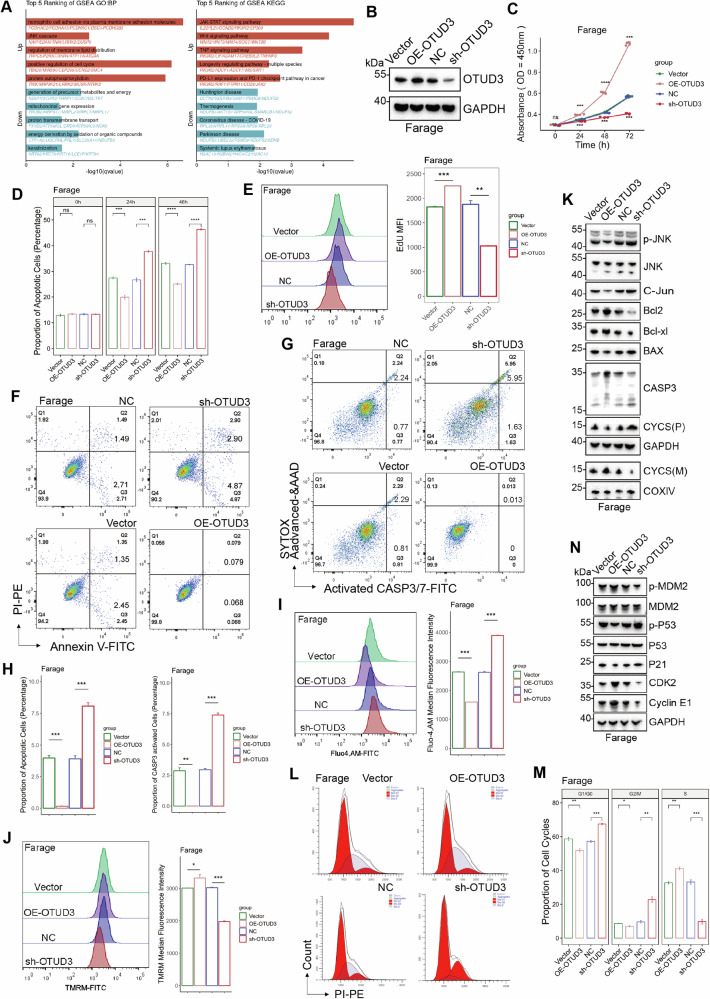
OTUD3 regulates the survival and cell cycle of DLBCL cells. **A** Gene Set Enrichment Analysis (GSEA) on the differentiation of OTUD3 expression levels in TCGA-DLBCL samples (top 33% vs. bottom 66%), top 5 ranking enriched termed were shown. **B** Expression level of OTUD3 in constructed stable Farage cells(Vector, OE-OTUD3, NC, sh-OTUD3). **C** CCK-8 assay on stable Farage cells. **D** LDH assay on stable Farage cells. **E** EdU assay on stable Farage cells. **F** Apoptosis rate of cells in all groups. **G** Proportion of cells undergoing activation of CASP3 in all groups. **H** Statistical analysis of apoptosis and active CASP3 experiments. **I** Intracellular calcium ion concentrations in all groups. **J** Mitochondrial activity in all groups. **K** Expression levels of apoptotic regulatory proteins. **L**, **M** Cell cycle distribution in all groups. **N** Expression levels of cell cycle regulatory proteins. Error bars represent the mean (*n* = 3) ± S.D. *P < 0.05, **P < 0.01, ***P < 0.001, ****P < 0.0001.

Based on the analysis of combined GTEx (The Genotype-Tissue Expression project) and TCGA (The Cancer Genome Atlas Program) - DLBCL data, OTUD3 is highly expressed in cancerous tissues in contrast with healthy cells (Fig. S1A). Patients with high OTUD3 expression (in the top 33% of OTUD3 expression levels) had a shorter progression-free survival in contrast to those with low levels (HR = 10, *P* = 0.035) (Fig. S1B). Dividing patients into two groups based on OTUD3 expression levels (high vs low), GSEA analysis revealed a high OTUD3 level is related to the Wnt pathway and positive regulation of the cell cycle (Fig. 1A). Spearman correlation analysis showed that OTUD3 is positively correlated with several oncogenic genes and PD-L1 (Fig. S1E). Patient-derived protein data revealed that OTUD3 is highly expressed in DLBCL tumor tissues and is associated with elevated levels of MYL12A and PD-L1 (Fig. S1D). Steady cell lines using OTUD3 downregulation and upregulation lentivirus were developed in three different DLBCL cell lines, namely Farage, OCI-LY-1, and Su-DHL-4 to examine the role of OTUD3 in DLBCL cells, and the efficiency of genetic manipulation was verified using Western Blotting (WB) (Figs. 1B, S1F). The CCK-8 assay revealed that overexpressing OTUD3 increased B-cell lymphoma cell proliferation, whereas knocking down OTUD3 decreased B-cell lymphoma cell proliferation (Figs. 1C, S1G). In the LDH experiment, the rate of dead cells in the sh-OTUD3 group was significantly higher than in the Negative Control (NC) group, while it was significantly lower in the OE-OTUD3 cohort than in the empty vector (Vector) (Figs. 1D, S1H–I). Consistently, the sh-OTUD3 group showed significantly lower EdU median fluorescence Intensity (MFI) than the NC control group, whereas, in contrast to the Vector study, the OE-OTUD3 group had a substantial rise in EdU MFI (Figs. 1E, S1J–K). Overall, OTUD3 enhanced DLBCL cell growth in vitro.

### OTUD3 regulates apoptosis and cell cycle in DLBCL cells

The overexpression of OTUD3 inhibited apoptosis and CASP3/7 activity in DLBCL cells while knocking down OTUD3 promoted apoptosis and CASP3/7 activity in these cells (Figs. 1F–H, S1L–N, S2A–D).

OE-OTUD3 DLBCL cells had lower intracellular calcium ion concentrations compared to the Vector group while knocking down OTUD3 increased intracellular calcium ion concentrations in DLBCL cells (Figs. 1I, S2E). TMRM fluorescence signals were stronger in DLBCL cells of the OE-OTUD3 group and reduced accumulation of TMRM dye in the sh-OTUD3 group (Figs. 1J, S2F). These results suggest that OTUD3 contributes to maintaining the integrity of the mitochondrial membrane in DLBCL cells.

OTUD3 overexpression increased Bcl2 and Bcl-xl levels while decreasing BAX, p-JNK1/2/3, C-Jun, and Cleaved-caspase3; the effects were reversed by OTUD3 knockdown (Figs. 1K, S2G, H). We also segregated cytoplasm and mitochondria in DLBCL cells, noting higher mitochondrial CYCS in the OE-OTUD3 group versus the Vector group, with less cytoplasmic release (Figs. 1K, S2G, H). In contrast, the sh-OTUD3 group showed increased cytoplasmic CYCS release versus the NC group, with reduced mitochondrial presence. These findings collectively demonstrate OTUD3’s dependence on the mitochondrial apoptosis pathway to inhibit apoptosis in DLBCL cells. The results of the cell cycle analysis showed an increase in the G0/G1 phase in cells with OTUD3 knock-down (Figs. 1L, M, S2I–L). Subsequently, OTUD3 knock-down may promote P21 expression by enhancing p-MDM2 mediated P53 phosphorylation levels, further reducing the downstream expression of CDK2 and Cyclin E1, thereby forcing DLBCL cells to arrest in the G1/G0 phase (Figs. 1N, S3A).

### Rupatadine is an inhibitor of OTUD3 and inhibits the viability of DLBCL cells

**Fig. 2:**
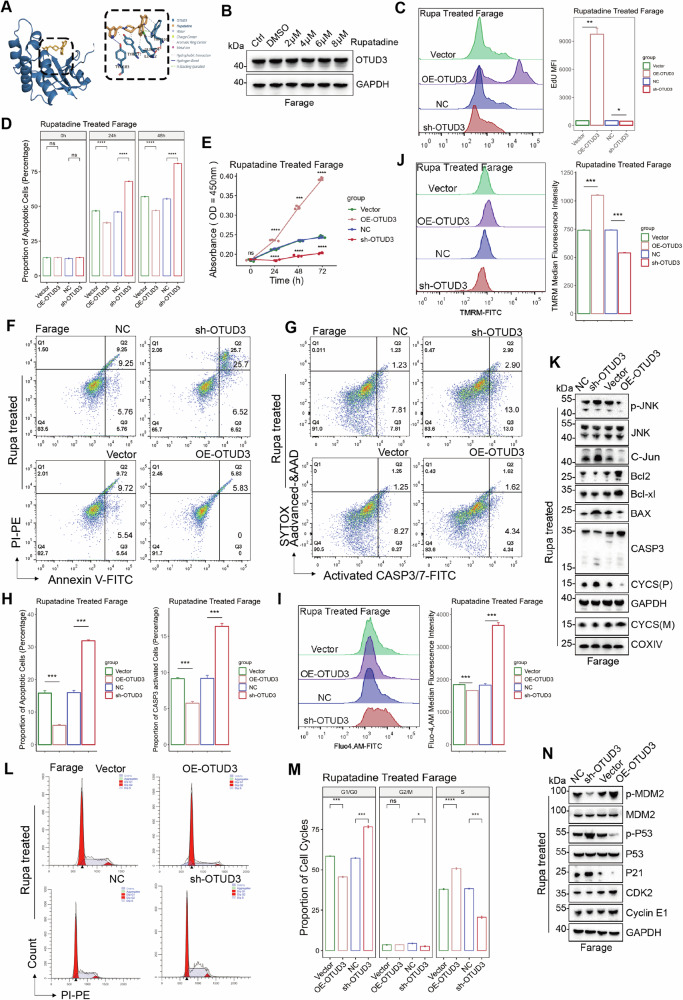
Rupatadine inhibits the survival and cell cycle of DLBCL cells. **A** Virtual docking analysis between the OTU domain of OTUD3 and Rupatadine(Rupa). **B** Expression level of OTUD3 under Rupa treatment. **C** EdU assay on Rupa treated stable Farage cells. **D** LDH assay on Rupa treated stable Farage cells. **E** CCK-8 assay on Rupa treated stable Farage cells. **F** Apoptosis rate of cells in all Rupa-treated groups. **G** Proportion of cells undergoing activation of CASP3 in all Rupa-treated groups. **H** Statistical analysis of apoptosis and active CASP3 experiments. **I** Intracellular calcium ion concentrations in all Rupa-treated groups. **J** Mitochondrial activity in all Rupa-treated groups. **K** Expression levels of apoptotic regulatory proteins under Rupa treatment. **L**, **M** Cell cycle distribution in all Rupa-treated groups. **N** Expression levels of cell cycle regulatory proteins under Rupa treatment. Error bars represent the mean (*n* = 3) ± S.D. *P < 0.05, **P < 0.01, ***P < 0.001, ****P < 0.0001.

Virtual docking using AutoDock for the OTU domain of OTUD3 and rupatadine (Rupa) revealed an estimated free energy of binding = −7.73 kcal/mol, suggesting a significant likelihood of direct binding between rupatadine and OTUD3 (Fig. 2A). The expression levels of OTUD3 in the DLBCL cell lines were assessed after the addition of rupatadine. However, rupatadine had little impact on the expression of OTUD3 (Figs. 2B, S3B).

We conducted IC50 experiments on the DLBCL cell lines, establishing and employing their respective IC50 values for subsequent treatments with rupatadine (Fig. S3C). Interestingly, rupatadine significantly inhibited the proliferative activity of all groups, especially the sh-OTUD3 group, in DLBCL cells (Figs. 2C–E, S3D–H). Either knocking down OTUD3 or treating with rupatadine can inhibit the proliferation of DLBCL cell lines (Figs. S3I, S8A, B). The addition of rupatadine intensifies apoptosis in all DLBCL cell groups and enhances the activity of CASP3/7 in all treated cells, with the sh-OTUD group exhibiting the most pronounced effects and the OE-OTUD3 group showing the least (Figs. 2F–H, S3J–M, S4A–D). Simultaneously, rupatadine increased the intracellular free calcium ions in all groups and diminished the mitochondrial activity of all treated cells (Figs. 2I, J, S4E–H). The trends in apoptosis protein alterations in all groups after rupatadine treatment are consistent with those without drug administration (Figs. 2K, S4I, J).

Coincidentally, rupatadine can induce G1/G0 phase arrest in DLBCL cells, with the sh-OTUD3 group showing the most pronounced effect and the OE-OTUD3 group showing the least (Figs. 2L, M, S4K–M). The trend of cell cycle regulatory proteins remains consistent with and without the addition of rupatadine (Figs. 2N, S4N, O).

These findings suggested that, while rupatadine does not affect the expression of OTUD3, it inhibits the survival of DLBCL cells through alternative mechanisms.

### OTUD3 maintains MYL12A stability

To investigate how OTUD3 influences DLBCL cell survival and cell cycle, immunoprecipitation and mass spectrometry were used to pinpoint potential OTUD3 interacting proteins (Fig. S7G). Notably, MYL12A, known to be linked with apoptosis, showed enhanced interaction with OTUD3 in Farage cells overexpressing OTUD3 versus controls.

**Fig. 3:**
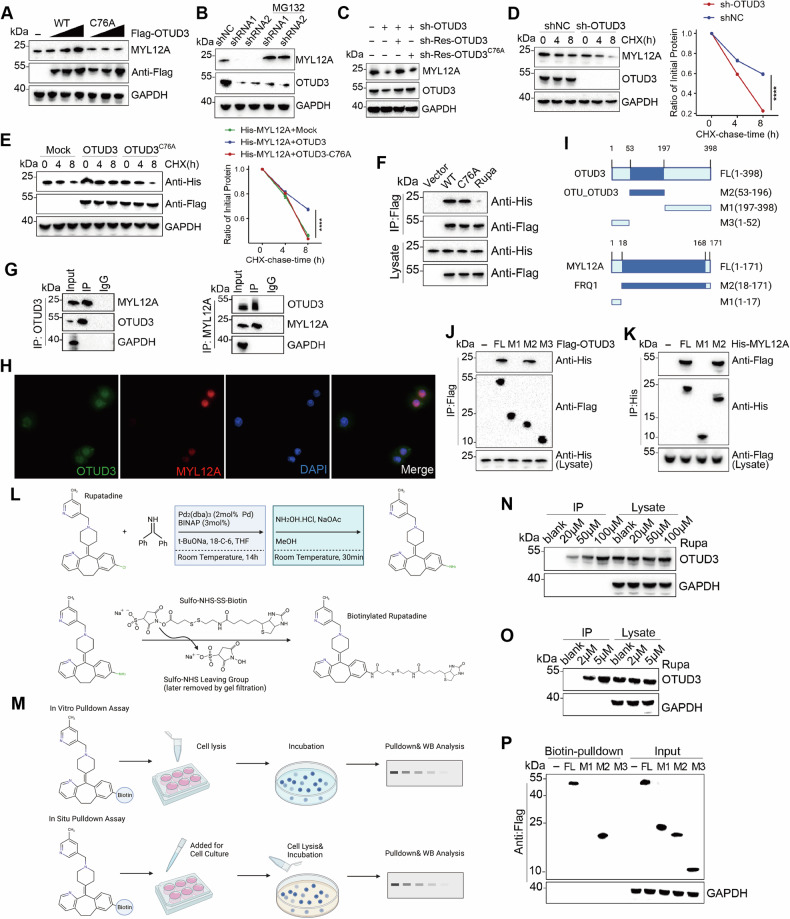
OTUD3 interacts with MYL12A and Rupatadine to stabilize MYL12A. **A** Flag-labeled wild-type (WT) OTUD3 or OTUD3-C76A was transfected into Farage cells, and cell lysates were analyzed by western blotting (WB) using anti-MYL12A antibody. **B** Farage cells were transfected with two independent OTUD3 shRNAs, followed by treatment with or without the proteasome inhibitor MG-132 (20 μM, 8 h), and subsequent analysis of OTUD3 and MYL12A was conducted. **C** In Farage cells transfected with OTUD3 sh-RNA, they were further transfected with Flag-tagged wild-type (WT) OTUD3 or OTUD3-C76A resistant to sh-RNA (sh-Res), WB analysis was performed for MYL12A levels. **D** In Farage cells stably expressing OTUD3 shRNA, quantitative analysis of MYL12A levels relative to GAPDH was demonstrated by WB after treatment with cycloheximide (CHX, 10 µg/ml). **E** Farage cells transfected with the specified plasmid were treated with CHX and collected at designated time points for WB, and the half-life of MYL12A was analyzed. **F** HEK293T cells were transfected with either His-MYL12A alone or in combination with Flag-tagged WT OTUD3 or OTUD3-C76A. Following immunoprecipitation (IP) with Flag beads from cell lysates, WB analysis was conducted using antibodies targeting His and Flag. (From left to right: Empty vector (Vector), OTUD3-WT, OTUD3-C76A, post-transfection with OTUD3-WT followed by Rupa treatment). **G** Co-immunoprecipitation (CO-IP) was used to confirm the interaction between OTUD3 and MYL12A in Farage cells. **H** Immunofluorescence revealed the colocalization of OTUD3 and MYL12A in Farage cells pre-fixed on glass slides. **I** Schematic representation of full-length (FL) OTUD3, MYL12A, and various deletion mutants. **J** HEK293T cells were co-transfected with His-MYL12A and Flag-tagged FL OTUD3 or deletion mutants. After immunoprecipitation with Flag beads from cell lysates, WB analysis was conducted using antibodies against His and Flag. **K** HEK293T cells were co-transfected with Flag-OTUD3 and His-tagged FL MYL12A or deletion mutants. After immunoprecipitation with His beads from cell lysates, WB analysis was performed using antibodies against Flag and His. **L** The synthetic pathway illustrates the process of substituting boronamine for the chlorine group on rupatadine and incorporating biotin labeling. **M** In vitro pulldown& WB: Biotin-pulldown and WB analysis performed after incubating biotinylated Rupatadine with Farage cell lysates. In situ pulldown & WB: Biotinylated Rupatadine was incubated with Farage cells, followed by cell lysis, biotin pulldown, and subsequent WB analysis. **N** WB analysis was conducted to assess the outcomes of the in vitro pulldown. **O** WB analysis was conducted to assess the outcomes of the in situ pulldown. **P** HEK293T cells were transfected with Flag-tagged FL OTUD3 or deletion mutants, followed by treatment with labeled rupatadine. After biotin-pulldown from cell lysates, WB analysis was conducted using antibodies against Flag. Error bars represent the mean (*n* = 3) ± S.D. *P < 0.05, **P < 0.01, ***P < 0.001, ****P < 0.0001.

Lin Yuan et al. previously identified OTUD3’s deubiquitinating role and its impairment in the C76A mutation, yet its functions in DLBCL remain unexplored. We transfected Farage cells with wild-type and C76A mutant OTUD3 plasmids, both flagged, to investigate OTUD3’s relationship with MYL12A. Wild-type (WT) OTUD3 increased MYL12A expression in a dose-dependent manner, suggesting it regulates MYL12A through its deubiquitinating activity (Fig. 3A). Knockdown of OTUD3 decreased MYL12A expression, which was mitigated by MG132 or siRNA-resistant wild-type OTUD3, but not by the C76A mutant (Fig. 3B, C).

Further, treating Farage cells with cycloheximide (CHX) showed that OTUD3 knockdown reduced MYL12A’s half-life, while overexpressing wild-type OTUD3, unlike the C76A variant, extended it (Fig. 3D, E). These findings indicate OTUD3’s role in stabilizing MYL12A.

### OTUD3 interacts with MYL12A and rupatadine

CO-IP experiments showed that in HEK293T cells, His-tagged MYL12A binds to both wild-type and C76A mutant OTUD3 with Flag tags. However, rupatadine reduced MYL12A’s binding to wild-type OTUD3 (Fig. 3F), indicating that while the C76A mutation doesn’t affect binding, rupatadine can disrupt their interaction. Further CO-IP experiments confirmed OTUD3 and MYL12A interaction in Farage cells, with immunofluorescence showing their colocalization (Fig. 3G, H).

To pinpoint binding domains, we used NCBI’s conserved domain prediction, then created and tested truncated mutants of both proteins. Results showed the M2 region of OTUD3 (amino acids 53–196) primarily interacts with MYL12A’s M2 region (amino acids 18–171, Fig. 3J, K).

We modified rupatadine by replacing its chlorine with an amino group and adding biotin, facilitating the exploration of its interaction with OTUD3 (Fig. 3L, M). Both in vitro and in situ pulldown showed rupatadine binding to OTUD3 in Farage cells dose-dependently (Fig. 3N, O). Biotin pulldown post-transfection in HEK293T cells further confirmed rupatadine binds to OTUD3’s M2, particularly the OTU domain (Fig. 3P). These results suggest rupatadine may compete with MYL12A for binding to OTUD3.

### OTUD3 deubiquitylates MYL12A

**Fig. 4:**
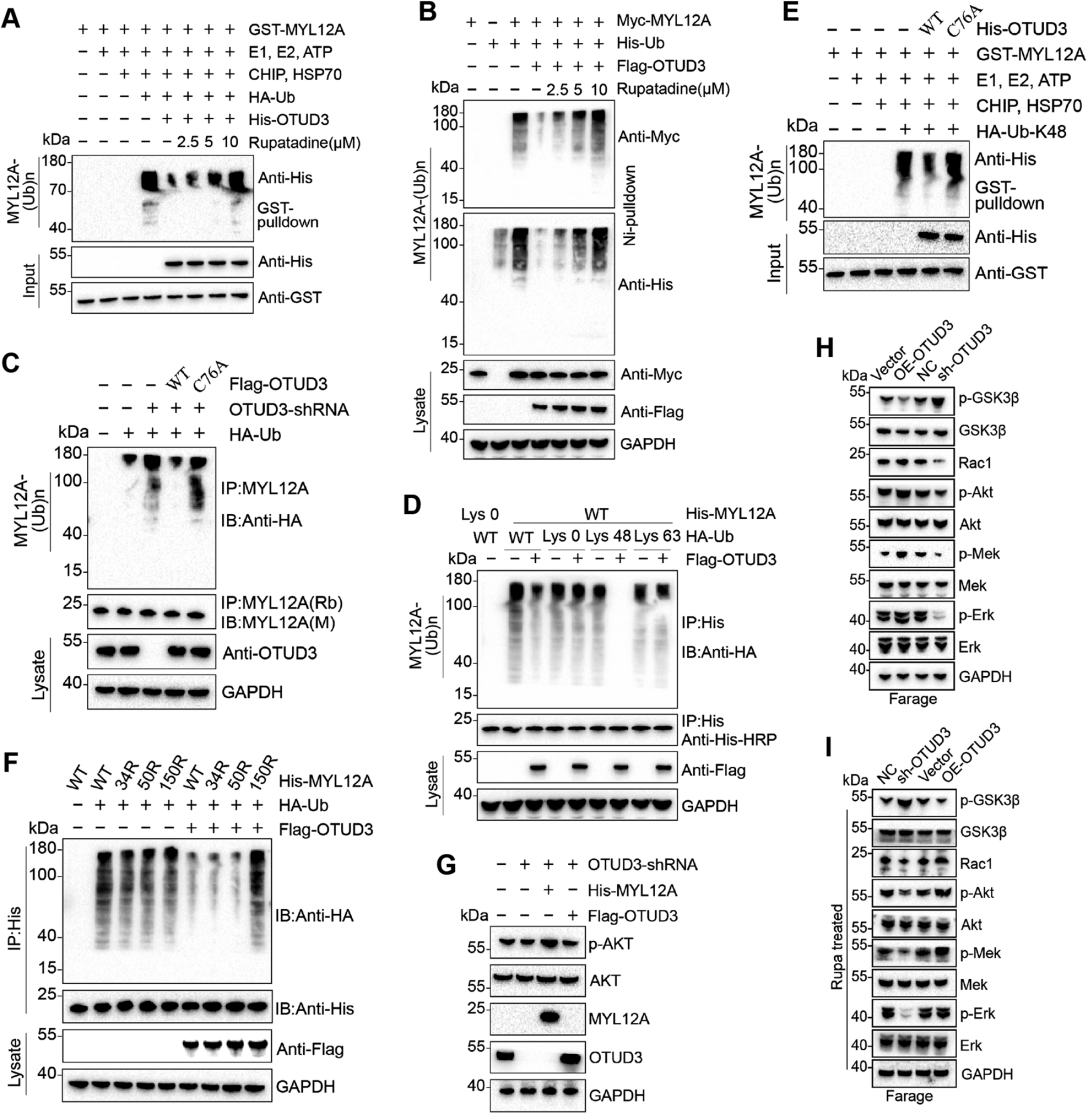
OTUD3 deubiquitylates MYL12A and activates Akt signaling. **A** Cell-free MYL12A ubiquitination experiment: GST-MYL12A and His-OTUD3 proteins, expressed and purified from bacteria, were incubated with commercial E1, UbE2D3 (E2), CHIP (E3), Hsp70, and ubiquitin at 37 °C for 2 h. The mixture underwent GST pulldown, and Western blot analysis was conducted using anti-His antibodies. **B** Farage cells were transfected with the specified constructs, followed by treatment with MG132 for 8 h before collection. Whole-cell lysates underwent Ni2+ beads pulldown, and Western blot analysis was performed using anti-Myc and anti-His antibodies to detect ubiquitinated MYL12A. **C** OTUD3 knock-down Stable Farage cells were co-transfected with Flag-OTUD3 WT or C76A mutant and HA-Ub. Cells were treated with MG132 (20 µM, 8 h) before collection. IP of cell lysates was performed using anti-MYL12A antibodies, followed by detection of MYL12A ubiquitination using anti-HA antibodies. **D** Farage cells were co-transfected with His-MYL12A, Flag-OTUD3, and plasmids encoding HA-Ub Lys0, Lys48-only, or Lys63-only. Subsequently, the ubiquitination of MYL12A was analyzed. **E** Cell-free ubiquitination experiments of MYL12A were performed using OTUD3 WT or C76A in conjunction with Lys48-only plasmids. **F** HEK293T cells were transfected with empty plasmid or Flag-OTUD3, HA-Ub, and either wild-type His-MYL12A or K-to-R mutant. Cell lysates underwent IP with anti-Flag beads, followed by immunoblot analysis using anti-HA or anti-Flag antibodies. **G** Plasmids encoding MYL12A or OTUD3 shRNA-resistant were transfected into MYL12A-knockout Farage cells with stable knockdown of OTUD3 using shRNA. Subsequently, analysis of Akt phosphorylation was conducted. **H**, **I** Protein expression in various components of the Akt pathway under normal conditions or after rupatadine treatment in all groups.

OTUD3, as a deubiquitinating enzyme, was tested for its ability to remove ubiquitin from MYL12A. In vitro experiments showed OTUD3 directly deubiquitinates MYL12A, with rupatadine dose-dependently inhibiting this activity (Fig. 4A). Exogenous wild-type OTUD3 reduced MYL12A ubiquitination levels more than the C76A mutant, and rupatadine modulation of this effect was also dose-dependent (Fig. 4B). Increased MYL12A ubiquitination from OTUD3 knockdown was lessened by wild-type OTUD3, not by the C76A mutant (Fig. 4C).

We then identified the specific ubiquitin chain affected by OTUD3; it preferentially removed Lys48-linked polyubiquitination, not monoubiquitination or Lys63-linked chains, which was confirmed in a cell-free system (Fig. 4D, E).

We created three lysine-arginine MYL12A mutants (K34R, K50R, K150R) based on predicted ubiquitination sites from GPS-Uber (http://gpsuber.biocuckoo.cn/wsresult.php). The K150 site had the highest prediction confidence. In CO-IP experiments, all mutants showed ubiquitination, but less so than wild-type MYL12A. With exogenous OTUD3, ubiquitination in the K150R mutant was similar to that in cells without exogenous OTUD3 but with wild-type MYL12A, while it significantly decreased in the other mutants, indicating OTUD3 deubiquitinates specifically at the K150 site, and the K150R mutation resists this action (Fig. 4F).

### OTUD3 and rupatadine regulate AKT via MYL12A to regulate DLBCL cell survival

In Farage cells with MYL12A knockout, manipulating OTUD3 altered total AKT protein and phosphorylation levels. Phosphorylation only increased when exogenous MYL12A was introduced, suggesting OTUD3’s dependency on MYL12A for regulating AKT phosphorylation (Fig. 4G). OTUD3 overexpression increased Rac1, p-Akt, p-Mek1/2, and p-Erk1/2 levels, but decreased p-GSK3β in the absence of rupatadine (Figs. 4H, S5O). Conversely, OTUD3 knockdown reduced Rac1, p-Akt, p-Mek1/2, and p-Erk1/2 levels while increasing p-GSK3β, maintaining consistent trends even with rupatadine treatment (Figs. 4I, S6A).

**Fig. 5:**
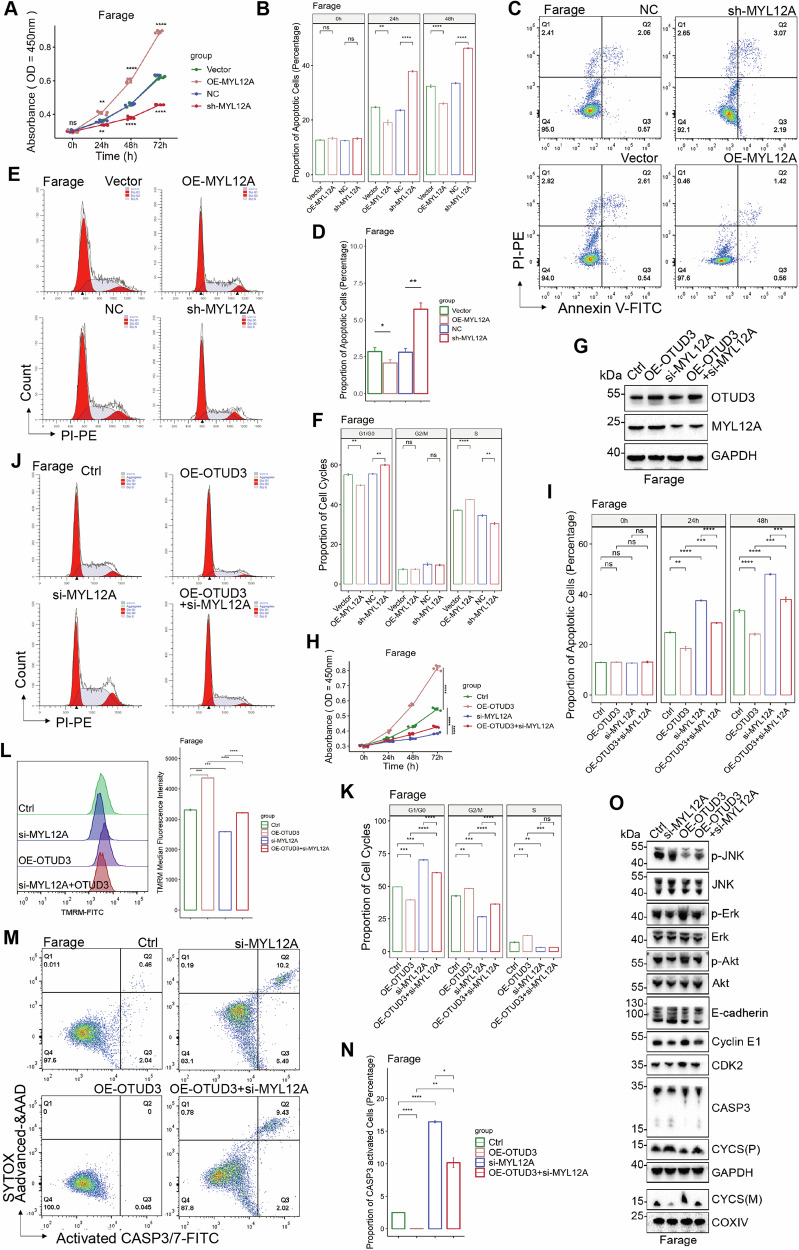
OTUD3 regulates the survival and cell cycle of DLBCL cells via MYL12A. **A** CCK-8 assay on stable Farage cells(Vector, OE-MYL12A, NC, sh-MYL12A). **B** LDH assay on stable Farage cells. **C**, **D** Apoptosis rate of cells in all groups. **E**, **F** Cell cycle distribution of stable Farage cells. **G** Expression level of OTUD3 and MYL12A in constructed stable Farage cells (Ctrl, OE-OTUD3, si-MYL12A, OE-OTUD3+si-MYL12A). **H** CCK-8 assay on transfected Farage cells (Ctrl, OE-OTUD3, si-MYL12A, OE-OTUD3+si-MYL12A) **I** LDH assay on transfected Farage cells. **J**, **K** Cell cycle distribution of transfected Farage cells. **L** Mitochondrial activity of transfected Farage cells. **M**, **N** Proportion of cells undergoing activation of CASP3 of transfected Farage cells. **O** Expression level of apoptotic regulatory proteins, cell cycle regulatory proteins, and Akt pathway proteins. Error bars represent the mean (*n* = 3) ± S.D. *P < 0.05, **P < 0.01, ***P < 0.001, ****P < 0.0001.

We have established stable DLBCL cell lines with OTUD3 stably overexpressed and MYL12A transiently knocked-down and verified their efficiency (Fig. S5A). Experiments on proliferation, apoptosis, and the cell cycle after manipulating MYL12A demonstrated that knocking down MYL12A similarly led to G1/G0 phase arrest and decreased survival capability in DLBCL cells (Figs. 5A–F, S5B–G). From this, it can be observed that MYL12A functions as an oncogene in DLBCL.

Simultaneous OTUD3 overexpression and MYL12A knockdown in DLBCL cells led to partial G1/G0 phase arrest, slower proliferation, increased CASP3 activity, and reduced mitochondrial activity versus control (Figs. 5G–N, S5H–M). Protein phosphorylation levels of AKT and ERK were intermediate between the OTUD3-overexpressed and MYL12A-silenced groups (Figs. 5O, S5N). Trends in apoptosis and cell cycle protein changes aligned with cellular findings, with MYL12A reduction lessening OTUD3’s survival advantages in DLBCL cells.

### OTUD3 and rupatadine regulate DLBCL cell survival and metastasis in vivo

**Fig. 6:**
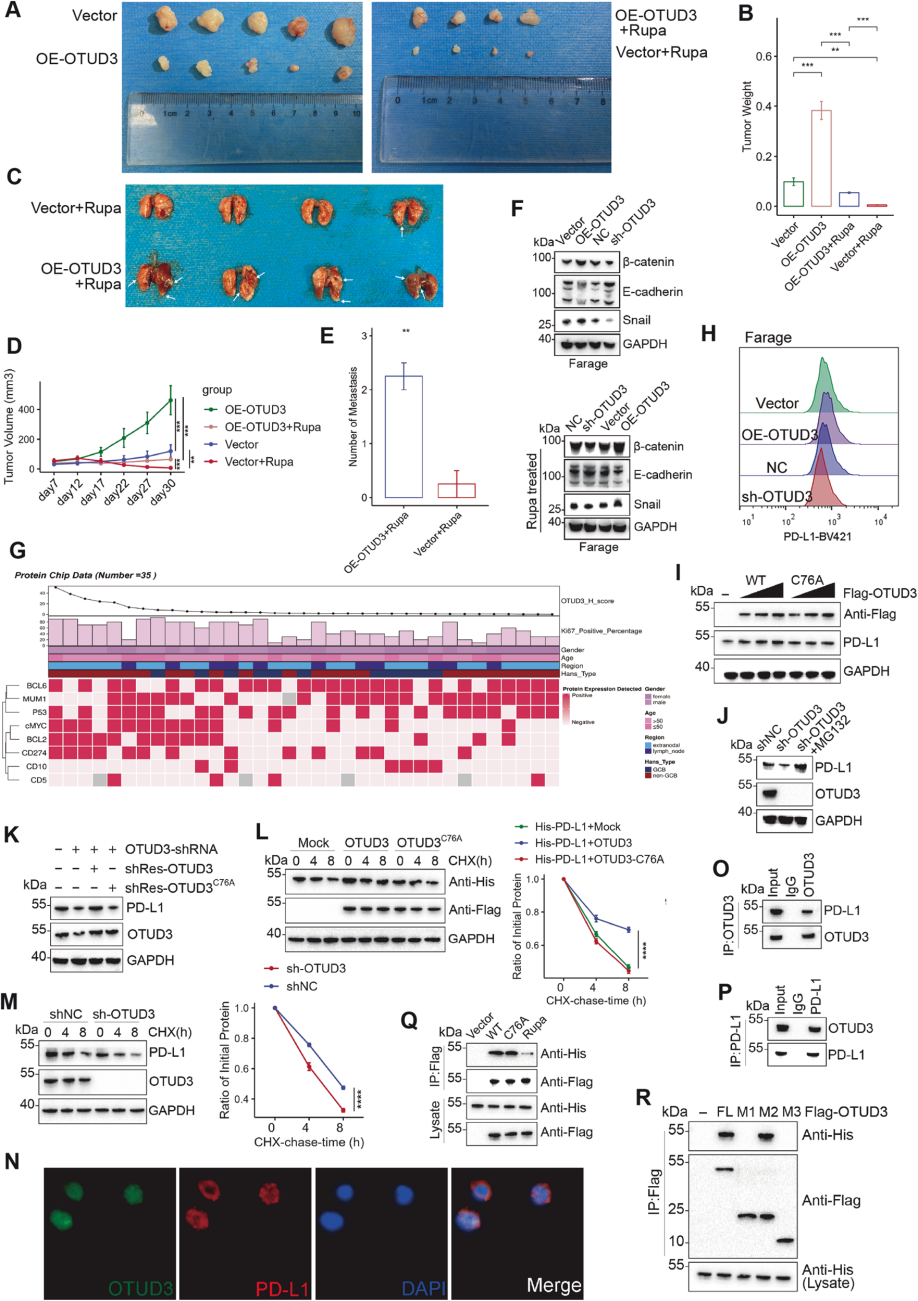
Rupatadine suppresses tumorgenesis and metastasis while OTUD3 stabilizes PD-L1. **A** Farage thriven Xenografted tumors in normal condition (left) or after rupa treatment (right). **B**, **D** Quantitative analysis of Xenografted tumor volume and weight. **C**, **E** mouse lungs from Farage thriven tumor metastasis model after rupa treatment and quantitative analysis of metastasis. **F** Expression level of β-catenin, E-cadherin, and snail proteins in stable Farage cells(Vector, OE-OTUD3, NC, sh-OTUD3). **G** Selected genes(Ki67, BCL6, MUM1, P53, cMYC, BCL2, CD274, CD10, CD5) were detected in protein chips of DLBCL patients’ tissues. **H** Surface PD-L1 expression of stable Farage cells. **I** Flag-labeled wild-type (WT) OTUD3 or OTUD3-C76A was transfected into Farage cells, and cell lysates were analyzed by WB using PD-L1 antibody. **J** Farage cells were transfected with OTUD3 shRNA, followed by treatment with or without MG-132 (20 μM, 8 h), and subsequent analysis of OTUD3 and PD-L1 was conducted. **K** In Farage cells transfected with OTUD3 sh-RNA, they were further transfected with Flag-tagged wild-type (WT) OTUD3 or OTUD3-C76A resistant to sh-RNA (sh-Res), WB analysis was performed for PD-L1 levels. **L** Farage cells transfected with the specified plasmid were treated with CHX (10 µg/ml) and collected at designated time points for WB, and the half-life of PD-L1 was analyzed. **M** In Farage cells stably expressing OTUD3 shRNA, quantitative analysis of PD-L1 levels was demonstrated by WB after treatment with CHX (10 µg/ml). **N** Immunofluorescence revealed the colocalization of OTUD3 and PD-L1 in Farage cells pre-fixed on glass slides. **O**, **P** Co-immunoprecipitation (CO-IP) was used to confirm the interaction between OTUD3 and PD-L1 in Farage cells. **Q** HEK293T cells were transfected with either His-PD-L1 alone or in combination with Flag-tagged WT OTUD3 or OTUD3-C76A. Following immunoprecipitation with Flag beads from cell lysates, WB analysis was conducted using antibodies targeting His and Flag. (From left to right: Empty vector (Vector), OTUD3-WT, OTUD3-C76A, post-transfection with OTUD3-WT followed by Rupa treatment). **R** HEK293T cells were co-transfected with His-PD-L1 and Flag-tagged FL OTUD3 or deletion mutants. After immunoprecipitation with Flag beads from cell lysates, WB analysis was conducted using antibodies against His and Flag. Error bars represent the mean ± S.D. *P < 0.05, **P < 0.01, ***P < 0.001, ****P < 0.0001.

We implanted OE-OTUD3 and Vector Farage cells into the subcutaneous regions on opposite sides of nude mice (Vector cells on the left, OE-OTUD3 on the right). OTUD3 overexpression significantly increased tumor growth and weight in vivo (Figs. 6A, B, D, S7H, I). Results from in vivo subcutaneous tumor formation and subsequent rupatadine treatment mirrored in vitro findings, with rupatadine notably reducing tumor size in both groups.

In the tail vein lung metastasis model, mice treated with rupatadine showed increased lung metastases in the OE-OTUD3 group compared to the Vector group (Figs. 6C–E, S7J, K).

OTUD3 overexpression elevated β-catenin and Snail levels but reduced E-cadherin levels (Figs. 6F, S6C, D). Knocking down OTUD3 had the opposite effects, suggesting OTUD3 may promote DLBCL metastasis through β-catenin regulation. Protein changes remained consistent under rupatadine treatment (Fig. S6E, F).

### OTUD3 maintains PD-L1 stability

We confirmed selected genes on tissue microarrays following prior bioinformatics analysis. Samples with elevated OTUD3 levels (higher H-score) showed increased proportions of KI67-positive cells and higher detection of cMYC, Bcl2, and CD274 proteins (Fig. 6G). We then assessed PD-L1 expression on Farage cells, establishing that OTUD3 modulation affects PD-L1 levels (Fig. 6H), suggesting OTUD3 might stabilize PD-L1.

WT OTUD3 increased PD-L1 expression dose-dependently, unlike the C76A mutation (Fig. 6I). OTUD3 knockdown in Farage cells decreased PD-L1 levels, which was reversed by MG132 or siRNA-resistant WT OTUD3, but not by the C76A mutant (Fig. 3J, K), indicating OTUD3’s role in stabilizing PD-L1.

Further evidence was provided by treating Farage cells with cycloheximide (CHX). OTUD3 knockdown shortened PD-L1’s half-life, whereas overexpressing WT OTUD3, not the C76A mutant, extended it (Fig. 6L, M). This supports that OTUD3 influences PD-L1 stability.

### OTUD3 interacts with and deubiquitylates PD-L1

Immunofluorescence showed OTUD3 and PD-L1 colocalization (Fig. 6N), and CO-IP experiments verified OTUD3’s interaction with MYL12A in Farage cells (Fig. 6O, P). In HEK293T cells, His-tagged PD-L1 bound both wild-type and C76A mutant OTUD3 with Flag tags (Fig. 6Q), and like MYL12A, PD-L1’s binding to wild-type OTUD3 decreased with rupatadine, indicating the C76A mutation doesn’t affect PD-L1 binding, but rupatadine can disrupt it.

Further CO-IP tests confirmed that OTUD3’s M2 region (amino acids 53–196), containing the OTU domain, facilitated its binding to PD-L1 (Fig. 6R).

**Fig. 7:**
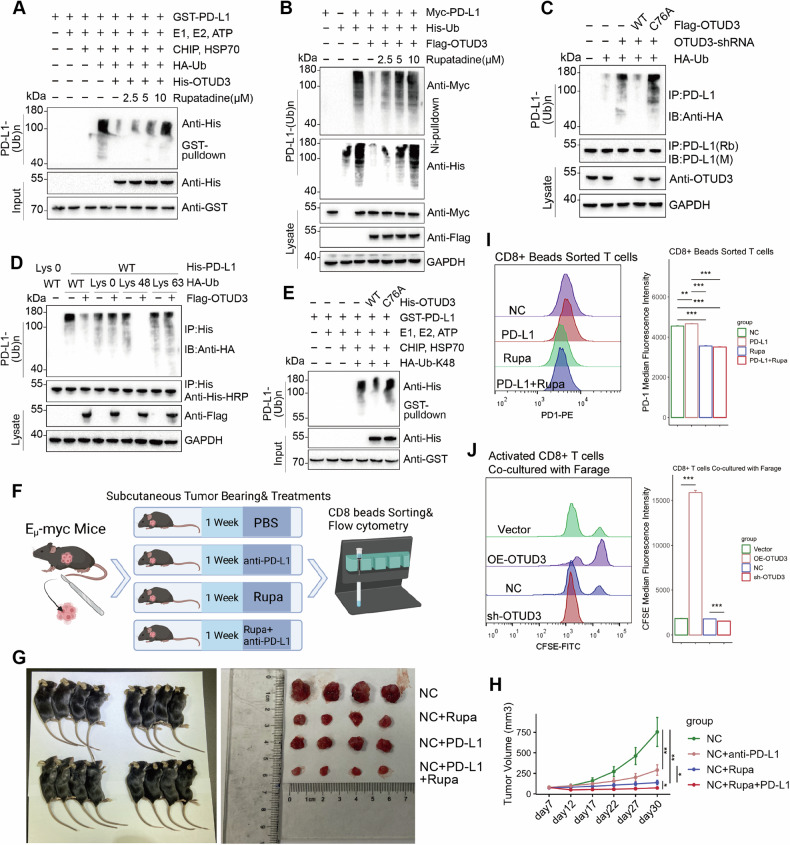
OTUD3 deubiquitylates PD-L1 and suppresses anti-tumor immunity. **A** Cell-free PD-L1 ubiquitination experiment: GST-PD-L1 and His-OTUD3 proteins, expressed and purified from bacteria, were incubated with commercial E1, UbE2D3 (E2), CHIP (E3), Hsp70, and ubiquitin at 37 °C for 2 h. The mixture underwent GST pulldown, and Western blot analysis was conducted using anti-His antibodies. **B** Farage cells were transfected with the specified constructs, followed by treatment with MG132 for 8 h before collection. Whole-cell lysates underwent Ni2+ beads pulldown, and Western blot analysis was performed using anti-Myc and anti-His antibodies to detect ubiquitinated PD-L1. **C** OTUD3 knock-down Stable Farage cells were co-transfected with Flag-OTUD3 WT or C76A mutant and HA-Ub. Cells were treated with MG132 (20 µM, 8 h) before collection. IP of cell lysates was performed using anti-PD-L1 antibodies, followed by detection of MYL12A ubiquitination using anti-HA antibodies. **D** Farage cells were co-transfected with His-PD-L1, Flag-OTUD3, and plasmids encoding HA-Ub Lys0, Lys48-only, or Lys63-only. Subsequently, the ubiquitination of PD-L1 was analyzed. **E** Cell-free ubiquitination experiments of PD-L1 were performed using OTUD3 WT or C76A in conjunction with Lys48-only plasmids. **F** Lymphoma cells derived from E_µ_-myc mice were extracted, immortalized, and subsequently subcutaneously implanted into healthy mice. The tumor was extracted and digested after the sacrifice of mice, and CD8+ T cells were isolated through magnetic bead separation using CD8+ beads. **G**, **H** Subcutaneous tumor volume after various treatments and quantitative analysis of tumor volume. **I** Surface PD-1 expression of CD8+ T cells from extracted and digested tumors after various treatments. **J** CFSE staining of CD3/CD28 beads activated CD8+ T cells co-cultured with Farage stable cells. Error bars represent the mean ± S.D. *P < 0.05, **P < 0.01, ***P < 0.001, ****P < 0.0001.

We then tested OTUD3’s ability to deubiquitinate PD-L1. In vitro results showed OTUD3 directly removed ubiquitin from PD-L1, with rupatadine inhibiting this activity dose-dependently (Fig. 7A). Wild-type OTUD3 reduced PD-L1 ubiquitination more than the C76A mutant, and rupatadine similarly moderated this effect (Fig. 7B). Increased PD-L1 ubiquitination from OTUD3 knockdown was lessened by wild-type OTUD3, not by the C76A mutant (Fig. 7C).

Finally, we determined that OTUD3 preferentially removed Lys48-linked polyubiquitination from PD-L1, confirmed in a cell-free system, rather than monoubiquitination or Lys63-linked chains (Fig. 7D, E).

### OTUD3 and rupatadine regulate T-cell exhaustion and anti-tumor immunity

Building on previous findings, we explored rupatadine’s potential to enhance anti-tumor immunity in DLBCL by transplanting tumors from Eµ-myc lymphoma mice subcutaneously into healthy mice, and treating them with PD-L1 monoclonal antibodies, rupatadine, or both (Fig. 7F). The rupatadine group saw a greater reduction in tumor volume than the PD-L1 antibody group, with combined treatments showing the most significant effects (Fig. 7G, H). Post-treatment, mouse tumors were dissected, and CD8+ T cells isolated; rupatadine treatment reduced PD-1 expression in these cells, suggesting it may prevent CD8+ T cell exhaustion and boost anti-tumor immunity (Fig. 7I).

**Fig. 8:**
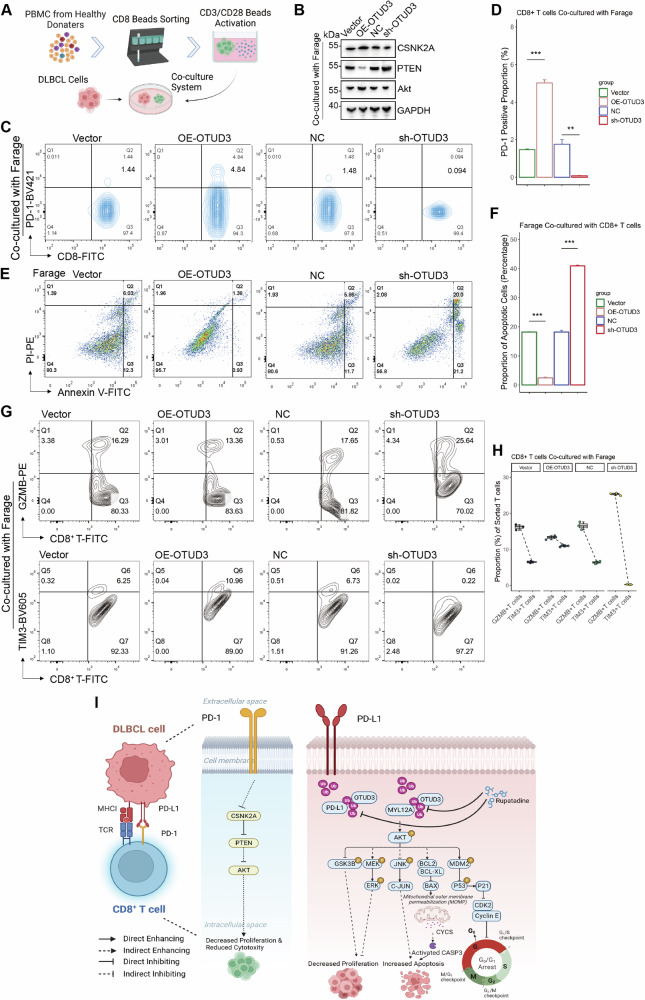
OTUD3 promotes the exhaustion of CD8+ T cells in the environment to evade immune responses. **A** CD8+ T cells were isolated, activated, and co-cultured with DLBCL cells. **B** Expression level of CSNK2A, PTEN, and Akt proteins in CD8+ T cells after co-culturing with Farage stable cells. **C**, **D** Proportion of CD8+, PD-1+ exhausted T cells after co-culturing with Farage stable cells. **E**, **F** Cytotoxicity assay with activated CD8+ T cells for Farage stable cells. **G**, **H** Proportion of CD8+, TIM3+ exhausted T cells and CD8+, GZMB+ effector T cells in sorted T cells after co-culturing with Farage stable cells. **I** The mechanistic scheme of this study. Error bars represent the mean (*n* = 3) ± S.D. *P < 0.05, **P < 0.01, ***P < 0.001, ****P < 0.0001.

Further experiments involved activating CD8+ T cells from healthy human PBMCs and co-culturing them with DLBCL cells to observe the effects of OTUD3 expression (Fig. 8A). T cells co-cultured with OTUD3-overexpressing cells showed decreased proliferation, while those with OTUD3 knockdown displayed increased proliferation and reduced exhaustion markers like PD-1 (Figs. 7J, 8C, D, S6D, E, G). Proteomic analysis post-co-culture revealed increased CSNK2A and AKT and decreased PTEN in T cells from the OTUD3 overexpression group, and the opposite in the knockdown group, indicating OTUD3’s role in T-cell exhaustion via the PD-L1-PD-1 pathway (Figs. 8B, S6F).

In addition, DLBCL cells showed varying apoptosis levels post co-culture, with the highest in the sh-OTUD3 group and the lowest in the OE-OTUD3 group (Figs. 8E, F, S6H–K). Co-culture results confirmed increased TIM3+ exhausted and decreased GZMB + T cells in the OE-OTUD3 group, with inverse trends in the sh-OTUD3 group (Figs. 8G, H, S7A, B).

In summary, OTUD3 modulates MYL12A stability, enhancing DLBCL survival and metastasis, and stabilizes PD-L1, promoting CD8+ T cell exhaustion and dampening tumor immunity (Fig. 8I).

## Discussion

In this study, we identified the oncogenic function of the deubiquitinase enzyme OTUD3 in DLBCL, noting increased OTUD3 expression in tumor tissues and its role in promoting DLBCL proliferation and metastasis both in vivo and in vitro. OTUD3 drives DLBCL progression by enhancing cell proliferation, blocking mitochondrial apoptosis, regulating the cell cycle, and facilitating metastasis. It activates key pathways like Akt-MAPK and MDM2-P53-P21 and interacts directly with MYL12A for deubiquitylation. In addition, we found that rupatadine, a common antiallergic drug, counters OTUD3’s effects by directly interacting with it. Importantly, we found that OTUD3’s interaction with PD-L1 contributes to CD8+ T cell exhaustion.

R-CHOP serves as the standard treatment regimen for newly diagnosed DLBCL patients. However, for refractory or relapsed cases, their prognosis is typically grim [18–20]. The primary challenge in treating DLBCL development is the resistance to standard therapies [21]. This resistance can be attributed to a myriad of factors, including genetic mutations, alterations in signaling pathways, and immune evasion mechanisms [22]. Identifying effective therapeutic targets for these patients remains a substantial challenge [23].

OTUD3 is a primarily cytoplasmic deubiquitinase, participating in numerous biological processes by modulating protein deubiquitination and protein stability [24, 25].

The significance of OTUD3 in malignancy is multifaceted and may be disease-specific. Early evidence suggests that OTUD3 functions as a deubiquitinase for PTEN, interacting with PTEN to enhance its stability, thus inhibiting the progression of breast cancer [8]. However, OTUD3 was found to interact with the glucose-regulated protein GRP78, enhancing its stability, and enhancing lung tumor proliferation and metastasis [6]. Undoubtedly, OTUD3 has consistently been linked to cancer progression and prognosis [7, 9]. Nonetheless, there is still a scarcity of research explaining the significance of OTUD3 in DLBCL.

Our findings in both in vitro and in vivo models suggest that OTUD3 promotes tumorigenesis in DLBCL. Through virtual docking applications, we identified an inhibitor of OTUD3, the common antiallergic drug rupatadine [26]. In in vitro and in vivo experiments, rupatadine suppressed the survival advantage and metastasis-promoting effects conferred by OTUD3 by competitive binding with OTUD3. Rupatadine operates to diminish the deubiquitination of MYL12A and PD-L1 by OTUD3.

Akt is frequently dysregulated in cancers, with phosphorylation-regulated Akt controlling the functions of downstream proteins involved in the survival and growth of cells, migration, and the cell cycle [27, 28] The MAPK pathway is a classic regulator of cancer development [29–31]. We observed that OTUD3 can depend on MYL12A to activate downstream components of the classical MAPK pathway, including Erk1/2 and JNK1/2/3 [32, 33]. It is likely that the activation of these downstream components further leads to the recruitment of cell cycle proteins, the activation of apoptosis-related proteins and the activation of metastasis-related proteins [34–38].

Researchers’ comprehension of the tumor microenvironment and tumor immune microenvironment has deepened [39–41]. Consequently, the demand for drug development, such as SHP2 inhibitors, which can simultaneously target both the tumor itself and immune checkpoints, is steadily increasing [42–44]. Through KEGG enrichment analysis based on expression levels of OTUD3, we discovered that the PD-L1 pathway is significantly enriched when OTUD3 expression is elevated. This finding was validated using tissue sourced from patients. Subsequently, we demonstrated that OTUD3 can modulate tumor immunity by stabilizing PD-L1. The PD-L1 pathway, as a component of the tumor’s adaptive immune resistance mechanism, interacts with the immune microenvironment and is reflected at the transcriptional landscape level. The correlation between the levels of OTUD3 expression and this change was captured and robustly validated by our research, proving the intricate relationship between OTUD3 and PD-L1—a valuable and challenging discovery. Targeting OTUD3 with rupatadine to simultaneously control DLBCL progression at both the tumor and T cell immune levels might be a promising choice, especially for elderly patients with limited tolerance [45, 46].

While considering the translational potential of our findings, it’s important to recognize several limitations. First, we used Farage, Su-DHL-4, and OCi-LY-1 cell lines, observing that the Farage line might tolerate rupatadine better, possibly due to its clumping growth habit affecting drug absorption; this requires further study. Second, although virtual docking and drug pulldown confirmed rupatadine’s interaction with OTUD3, the possibility of other targets influencing its effect on DLBCL remains. In addition, OTUD3’s role as a deubiquitinating enzyme significantly affects protein stability, and manipulating OTUD3 could impact various proteins under its regulation. This study particularly examined two molecules affecting phenotype but did not rule out other potential effects of OTUD3. Lastly, the absence of sufficient clinical data prevents us from comparing survival or immunotherapeutic efficacy in DLBCL patients treated with or without rupatadine, highlighting the need for further research.

## Conclusions

In our current research, we have unveiled the oncogenic role of OTUD3 in DLBCL and identified an effective drug, rupatadine, for OTUD3 inhibition. We have demonstrated that OTUD3 can directly bind to MYL12A and PD-L1, thereby influencing their ubiquitination levels and expression, subsequently facilitating the survival and immune evasion of DLBCL cells. These findings underscore the potential utility of rupatadine in targeting DLBCL and modulating the immune response.

### Supplementary information

supplementary Figures

Full and uncropped western blots

Antibody List

MS results

## Data Availability

All data were provided in the main text and supplementary materials of the article. Full and uncropped western blots were uploaded as supplementary files “WB negatives”.
