# Supplementary material for: Rupatadine-inhibited OTUD3 promotes DLBCL progression and immune evasion through deubiquitinating MYL12A and PD-L1
Source: Cell Death Dis. 2024 Aug 3;15(8):561. doi: 10.1038/s41419-024-06941-x (PMC11297949; doi:10.1038/s41419-024-06941-x)
Supplement: Supplementary file 1 — supplementary Figures [file 41419_2024_6941_MOESM1_ESM.pdf]

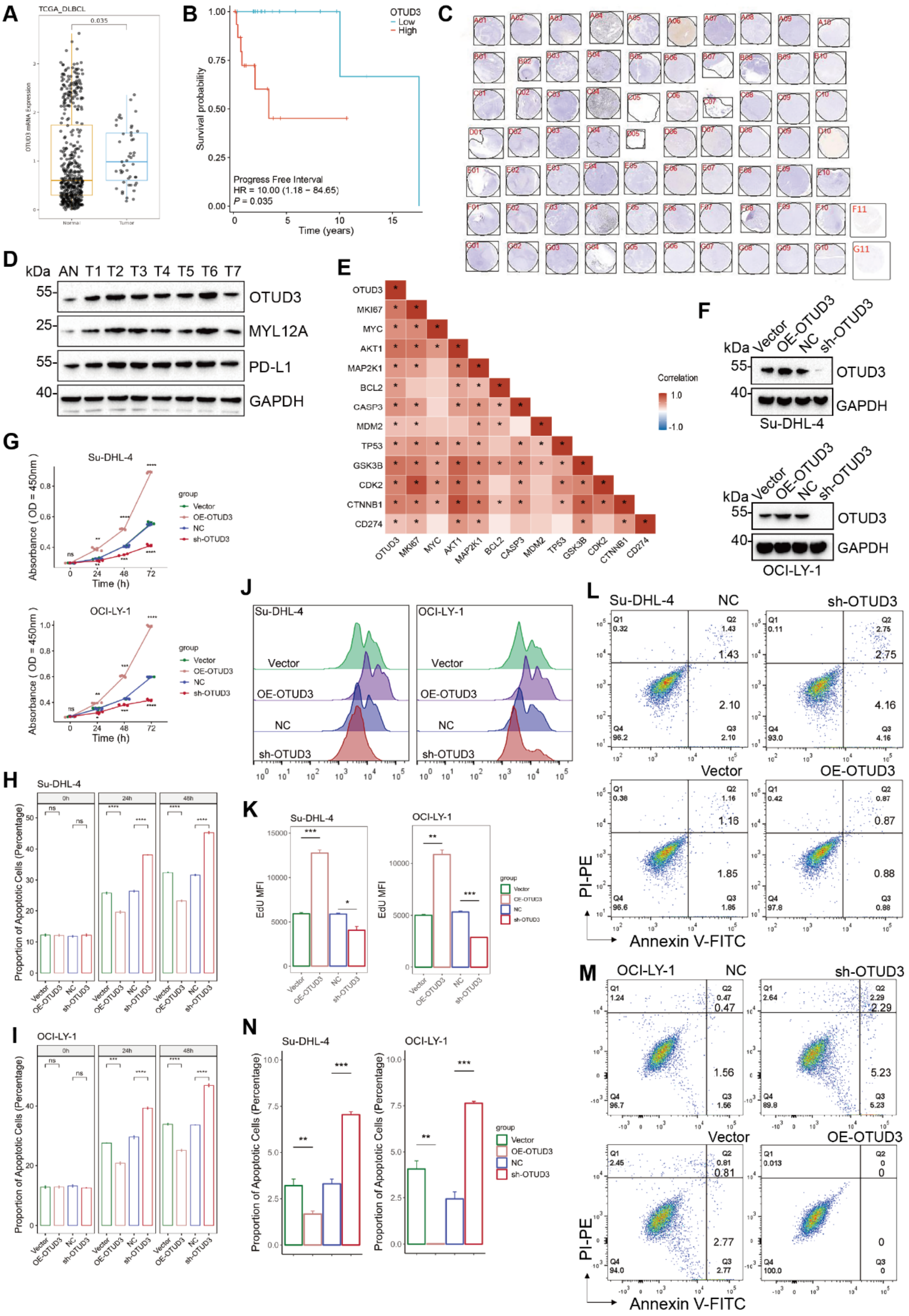

Fig.S1 OTUD3 regulates the survival of DLBCL cells A Expression of OTUD3 in TCGA-DLBCL samples and GTEx normal samples. **B** Survival analysis on the differentiation of OTUD3 expression levels in TCGA-DLBCL samples (top 33% vs. bottom 66%) **C** Detection of OTUD3 on protein chips. **D** WB of Patient-derived samples including tumor tissues and adjacent normal tissues. **E** Pearson correlation

analysis among OTUD3 and selected genes in TCGA-DLBCL samples. **F** Expression level of OTUD3 in constructed stable Su-DHL-4 and OCI-LY-1 cells(Vector, OE-OTUD3, NC, sh-OTUD3). **G** CCK-8 assay on stable Su-DHL-4 and OCI-LY-1 cells. **H, I** LDH assay on stable Su-DHL-4 and OCI-LY-1 cells. **J, K** EdU assay on stable Su-DHL-4 and OCI-LY-1 cells. **L-N** Apoptosis rate of stable Su-DHL-4 and OCI-LY-1 cells in all groups. Error bars represent the mean (n = 3) ±S.D. *P < 0.05, **P < 0.01, ***P < 0.001, ****P<0.0001.

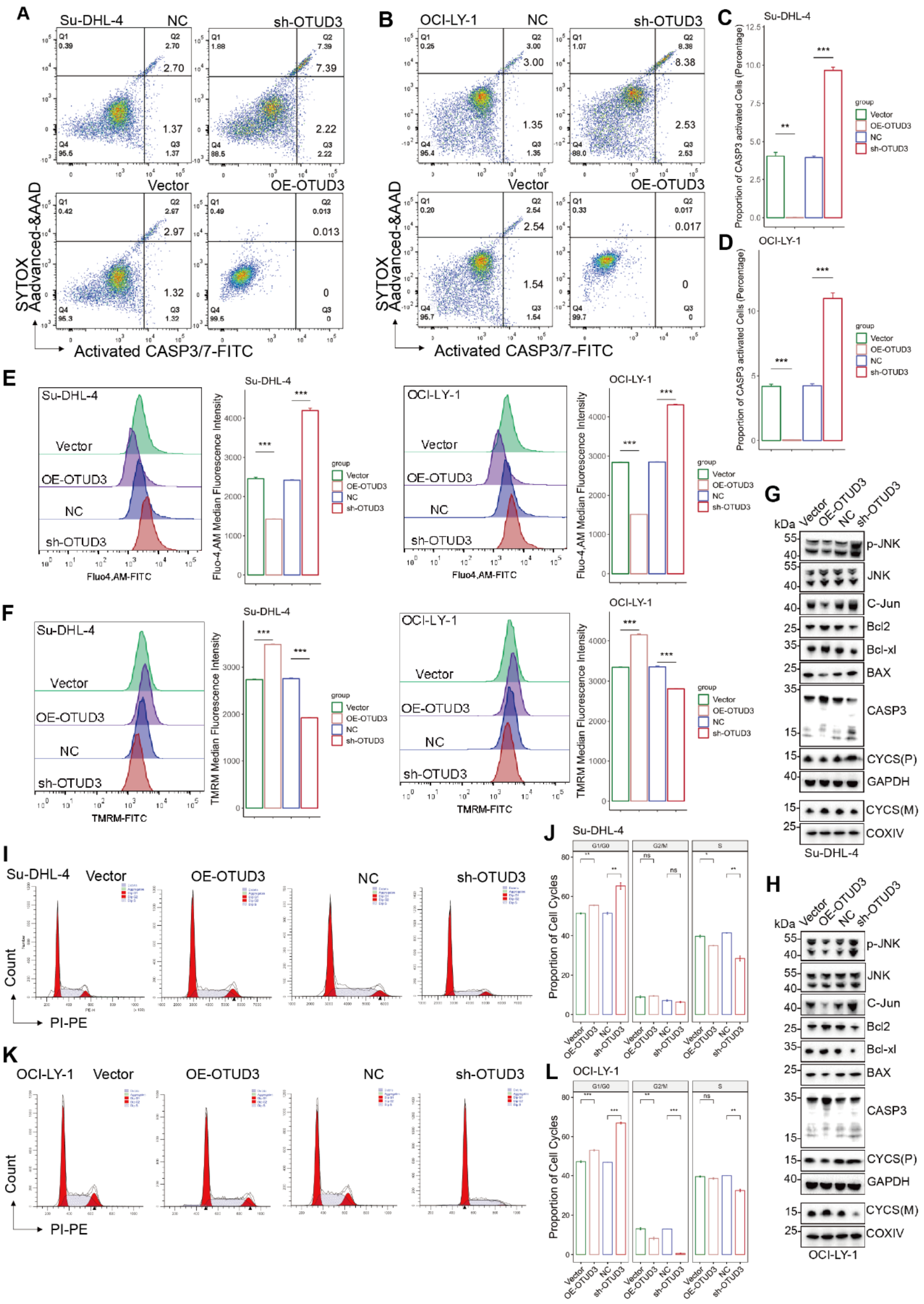

Fig.S2 OTUD3 regulates the apoptosis and cell cycle of DLBCL cells. A-D Proportion of Su-DHL-4 and OCI-LY-1 cells undergoing activation of CASP3 in all groups. **E** Intracellular calcium ion concentrations of Su-DHL-4 and OCI-LY-1 cells in all groups. **F** Mitochondrial activity of Su-DHL-4 and OCI-LY-1 cells in all groups. **G, H** Expression levels of apoptotic regulatory proteins in Su-DHL-4 and OCI-LY-1 cells. **I-L** Cell cycle distribution of Su-DHL-4 and OCI-LY-1 cells in all groups. Error bars represent the mean (n = 3) ±S.D. *P < 0.05, **P < 0.01, ***P < 0.001, ****P<0.0001.

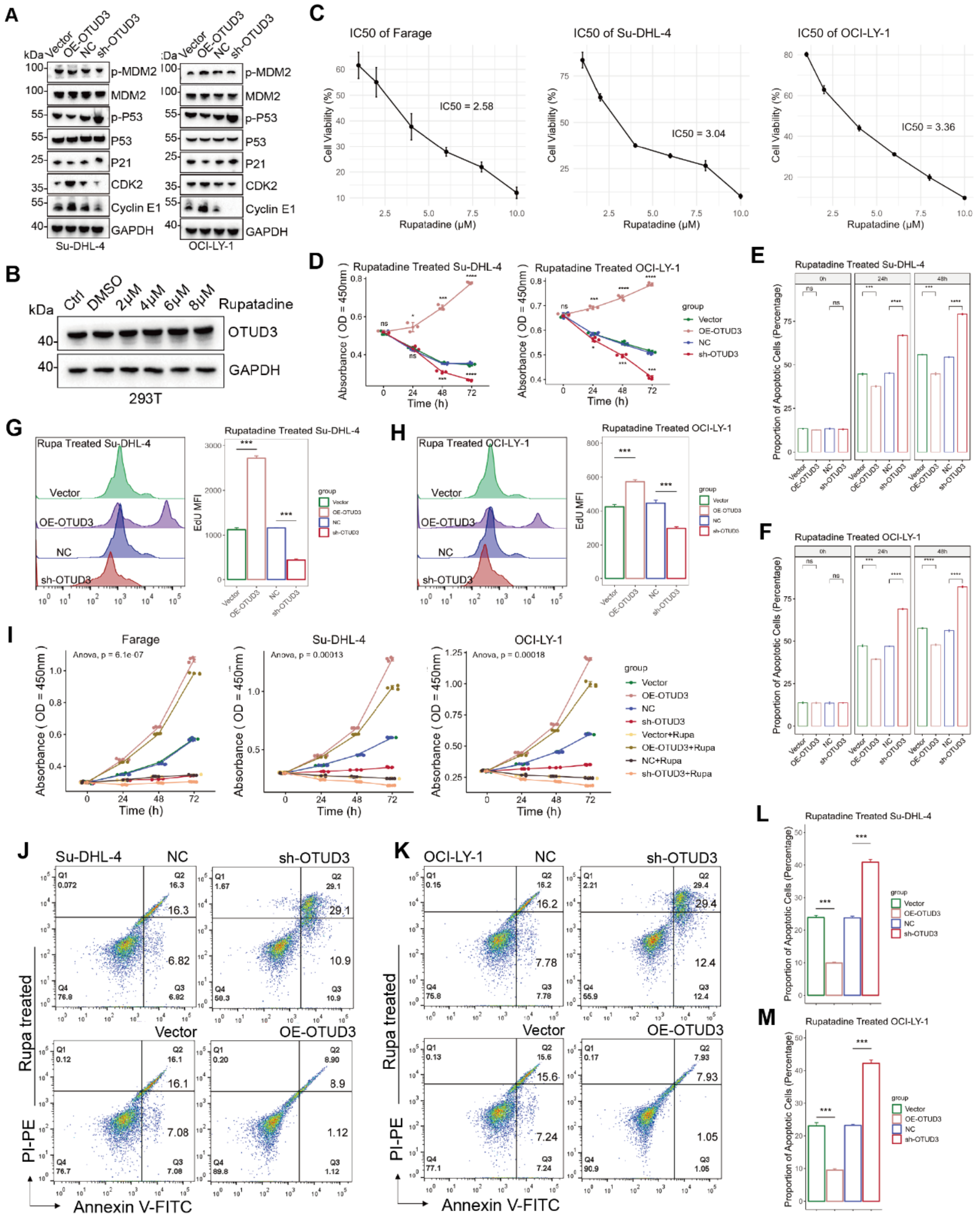

Fig.S3 Rupatadine regulates the survival of DLBCL cells. A Expression levels of cell cycle regulatory proteins in Su-DHL-4 and OCI-LY-1 cells. **B** Expression level of OTUD3 after rupatadine treatment. **C** IC50 assay on Rupa-treated Farage, Su-DHL-4 and OCI-LY-1 cells. **D** CCK-8 assay on Rupa-treated Su-DHL-

4 and OCI-LY-1 cells. **E, F** LDH assay on Rupa-treated Su-DHL-4 and OCI-LY-1 cells. **G, H** EdU assay on Rupa treated Su-DHL-4 and OCI-LY-1 cells. **I** CCK-8 assay on all groups. **J-M** Apoptosis rate of Rupa treated Su-DHL-4 and OCI-LY-1 cells in all groups. Error bars represent the mean (n = 3) ±S.D. *P < 0.05, **P < 0.01, ***P < 0.001, ****P<0.0001.

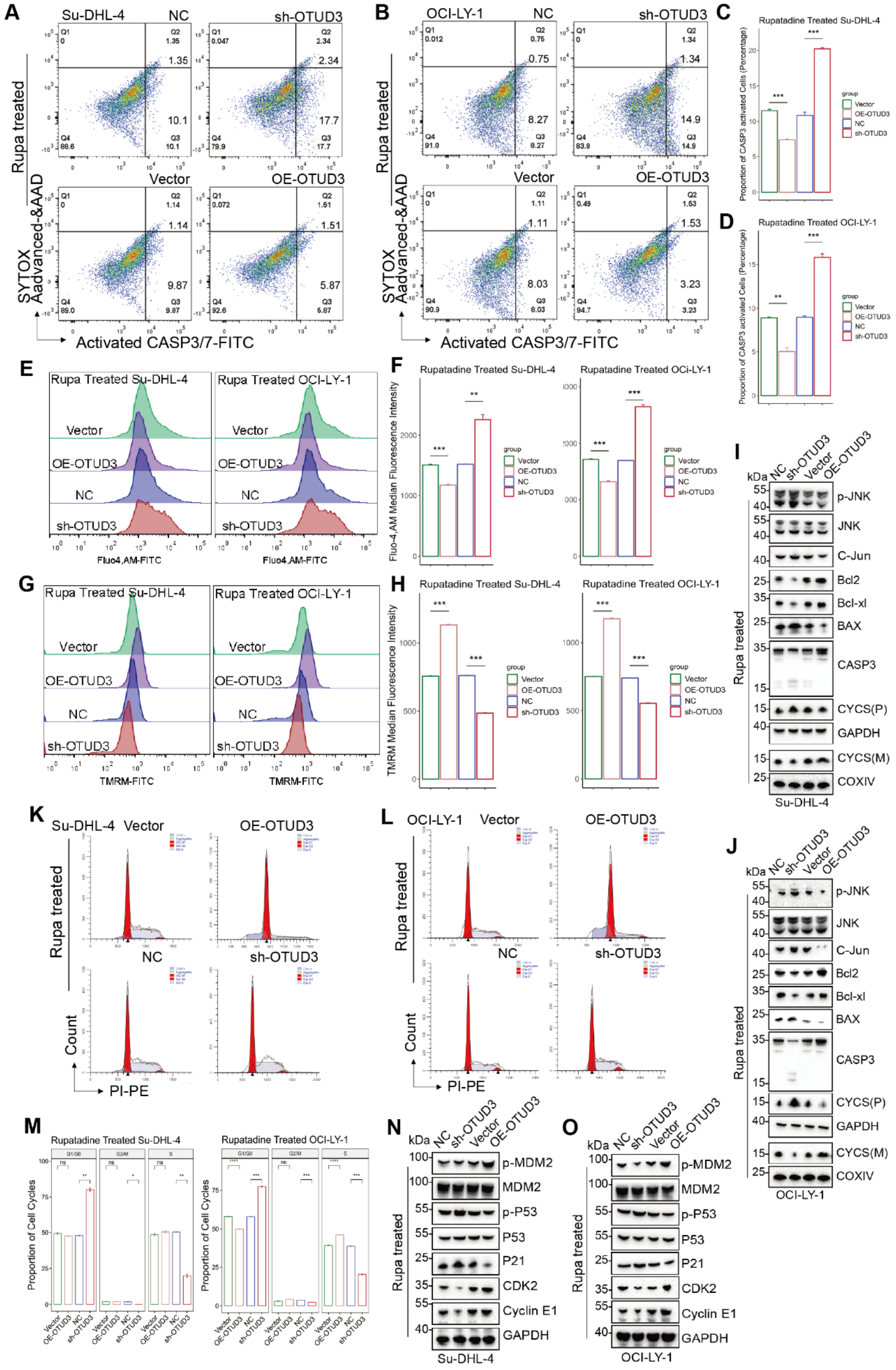

Fig.S4 Rupatadine regulates the apoptosis and cell cycle of DLBCL cells. A-D Proportion of cells undergoing activation of CASP3 in Rupa-treated Su-DHL-4 and OCI-LY-1 cells. **E, F** Intracellular calcium

ion concentrations of Rupa-treated Su-DHL-4 and OCI-LY-1 cells in all groups. **G, H** Mitochondrial activity of Rupa-treated Su-DHL-4 and OCI-LY-1 cells in all groups. **I, J** Expression levels of apoptotic regulatory proteins in Rupa treated Su-DHL-4 and OCI-LY-1 cells. **K-M** Cell cycle distribution of Rupa treated Su-DHL-4 and OCI-LY-1 cells in all groups. **N, O** Expression levels of cell cycle regulatory proteins in Rupa treated Su-DHL-4 and OCI-LY-1 cells. Error bars represent the mean (n = 3) ±S.D. *P < 0.05, **P < 0.01, ***P < 0.001, ****P<0.0001.

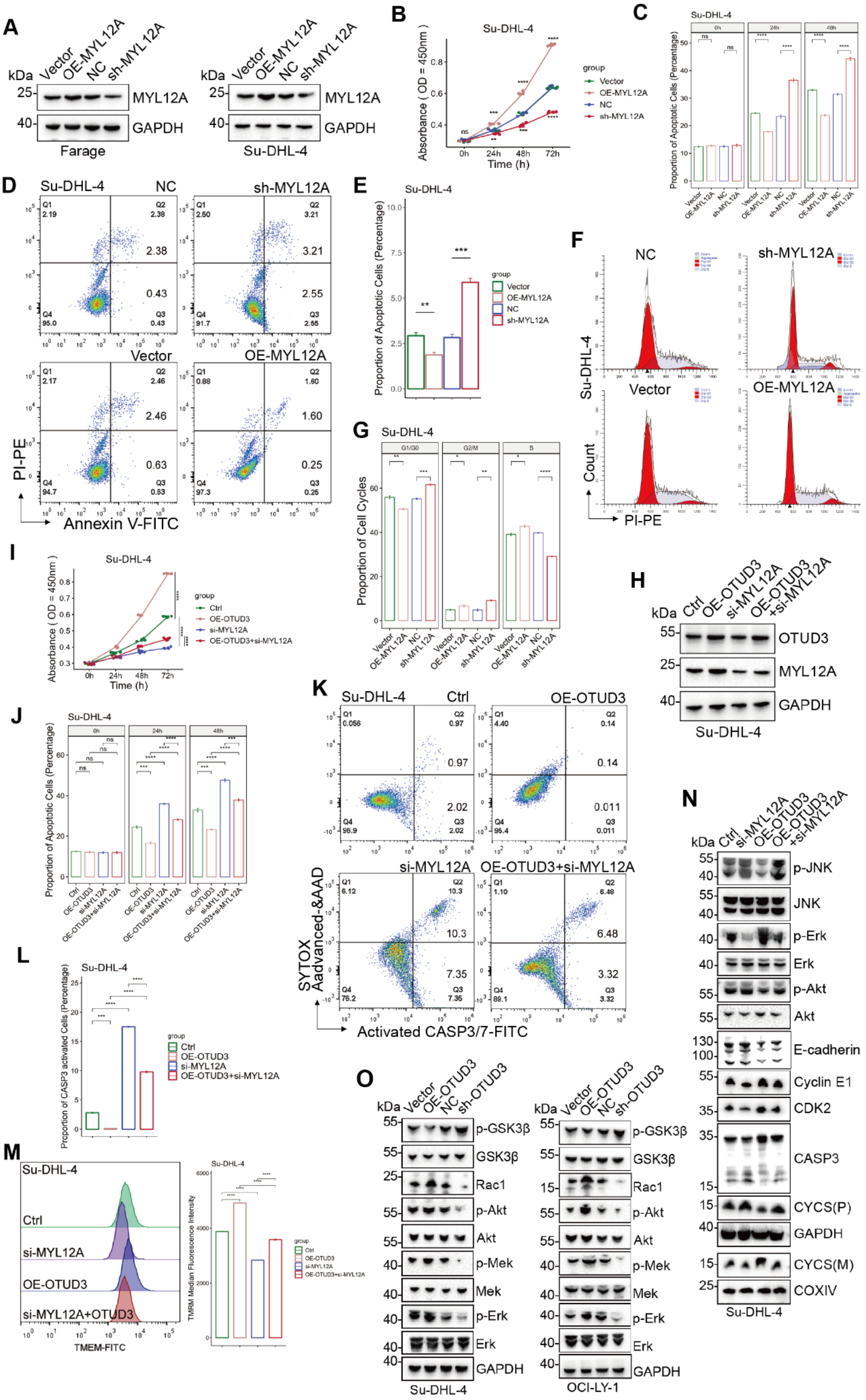

A
kDa
Vector
OE-MYL12A
NC
sh-MYL12A
25
40
MYL12A
GAPDH
Farage
Su-DHL-4
B
Su-DHL-4
Absorbance (OD = 450nm)
Time (h)
0h
24h
48h
72h
group
Vector
OE-MYL12A
NC
sh-MYL12A
C
Proportion of Apoptotic Cells (Percentage)
D
Su-DHL-4
NC
sh-MYL12A
Vector
OE-MYL12A
PI-PE
Annexin V-FITC
E
Proportion of Apoptotic Cells (Percentage)
F
Su-DHL-4
Count
PI-PE
G
Proportion of Cell Cycles
G1/G0
G2/M
S
H
Ctrl
OE-OTUD3
si-MYL12A
OE-OTUD3 +si-MYL12A
55
OTUD3
I
Ctrl
OE-OTUD3
si-MYL12A
OE-OTUD3+si-MYL12A
J
K
SYTOX Aadvanced-&AAD
Activated CASP3/7-FITC
L
Proportion of CASP3 activated Cells (Percentage)
M
TMEM-FITC
si-MYL12A+OTUD3
TMRM Median Fluorescence Intensity
N
p-JNK
JNK
p-Erk
Erk
p-Akt
Akt
E-cadherin
Cyclin E1
CDK2
CASP3
CYCS(P)
CYCS(M)
COXIV
O
sh-OTUD3
p-GSK3β
GSK3β
Rac1
p-Mek
Mek
OCI-LY-1

Fig.S5 OTUD3 regulates the survival and cell cycle of DLBCL cells via MYL12A A Expression level of MYL12A in constructed stable Farage and Su-DHL-4 cells(Vector, OE-MYL12A, NC, sh- MYL12A) **B** CCK-8 assay on stable Su-DHL-4 cells(Vector, OE-MYL12A, NC, sh-MYL12A). **C** LDH assay on stable Su-DHL-4 cells. **D, E** Apoptosis rate of cells in all groups. **F, G** Cell cycle distribution of stable Su-DHL-4 cells. **H** Expression level of OTUD3 and MYL12A in constructed stable Farage cells(Ctrl, OE-OTUD3, si-MYL12A, OE-OTUD3+si-MYL12A). **I** CCK-8 assay on transfected Su-DHL-4 cells(Ctrl, OE-OTUD3, si-MYL12A, OE-OTUD3+si-MYL12A) **J** LDH assay on transfected Su-DHL-4 cells. **K, L** Proportion of cells undergoing activation of CASP3 of transfected Su-DHL-4 cells. **M** Mitochondrial activity of transfected Su-DHL-4 cells. **N** Expression level of apoptotic regulatory proteins, cell cycle regulatory proteins, and Akt pathway proteins in Su-DHL-4 cells. **O** Protein expression in various components of the Akt pathway in Su-DHL-4 and OCI-LY-1 cells. Error bars represent the mean (n = 3) ±S.D. *P < 0.05, **P < 0.01, ***P < 0.001, ****P<0.0001.

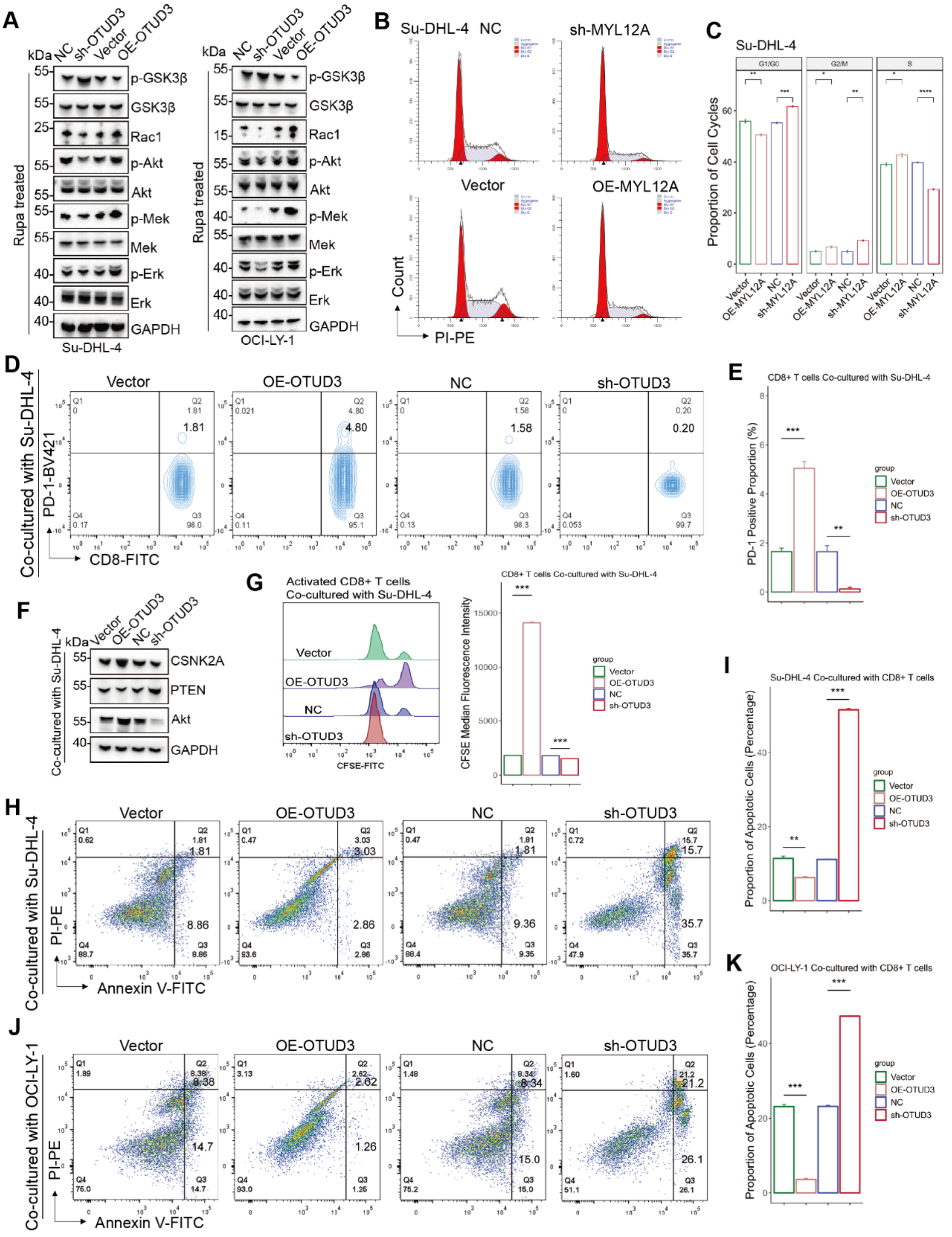

Fig.S6 OTUD3 promotes the exhaustion of CD8+ T cells in the environment to evade immune responses A Protein expression in various components of the Akt pathway after rupatadine treatment in Su-DHL-4 and OCI-LY-1 cells. B, C Cell cycle distribution of transfected Su-DHL-4 cells. D, E Proportion of CD8+, PD-1+ exhausted T cells after co-culturing with Su-DHL-4 stable cells. F Expression level of CSNK2A, PTEN, and Akt proteins in CD8+ T cells after co-culturing with Su-DHL-4 stable cells. G CFSE staining of CD3/CD28 beads activated CD8+ T cells co-cultured with Su-DHL-4 stable cells H-K Cytotoxicity assay with activated CD8+ T cells for Su-DHL-4 and OCI-LY-1 stable cells. Error bars represent the mean (n = 3) ±S.D. $*P < 0.05$, $**P < 0.01$, $***P < 0.001$, $****P<0.0001$.

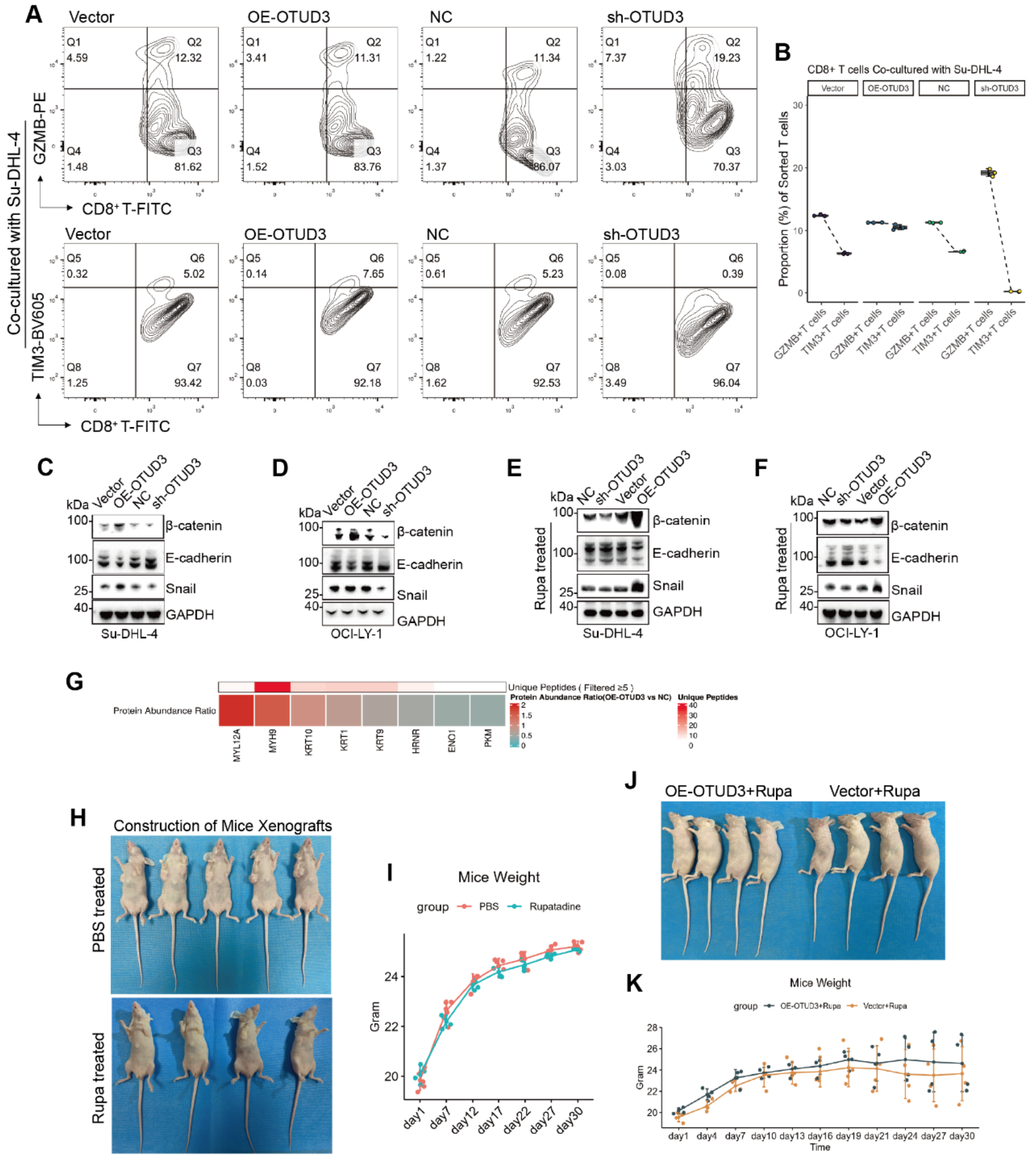

Fig.S7 A, B Proportion of CD8+, TIM3+ exhausted T cells and CD8+, GZMB+ effector T cells in sorted T cells after co-culturing with Su-DHL-4 stable cells. **C-F** Expression level of β-catenin, E-cadherin, and snail proteins in stable Su-DHL-4 and OCI-LY-1 cells with or without Rupa treatment. **G** The interactions of OTUD3 were identified using immunoprecipitation followed by mass spectrometry. **H, I** Condition and weight of mice subcutaneous tumor models. **J, K** Condition and weight of mice metastasis models.

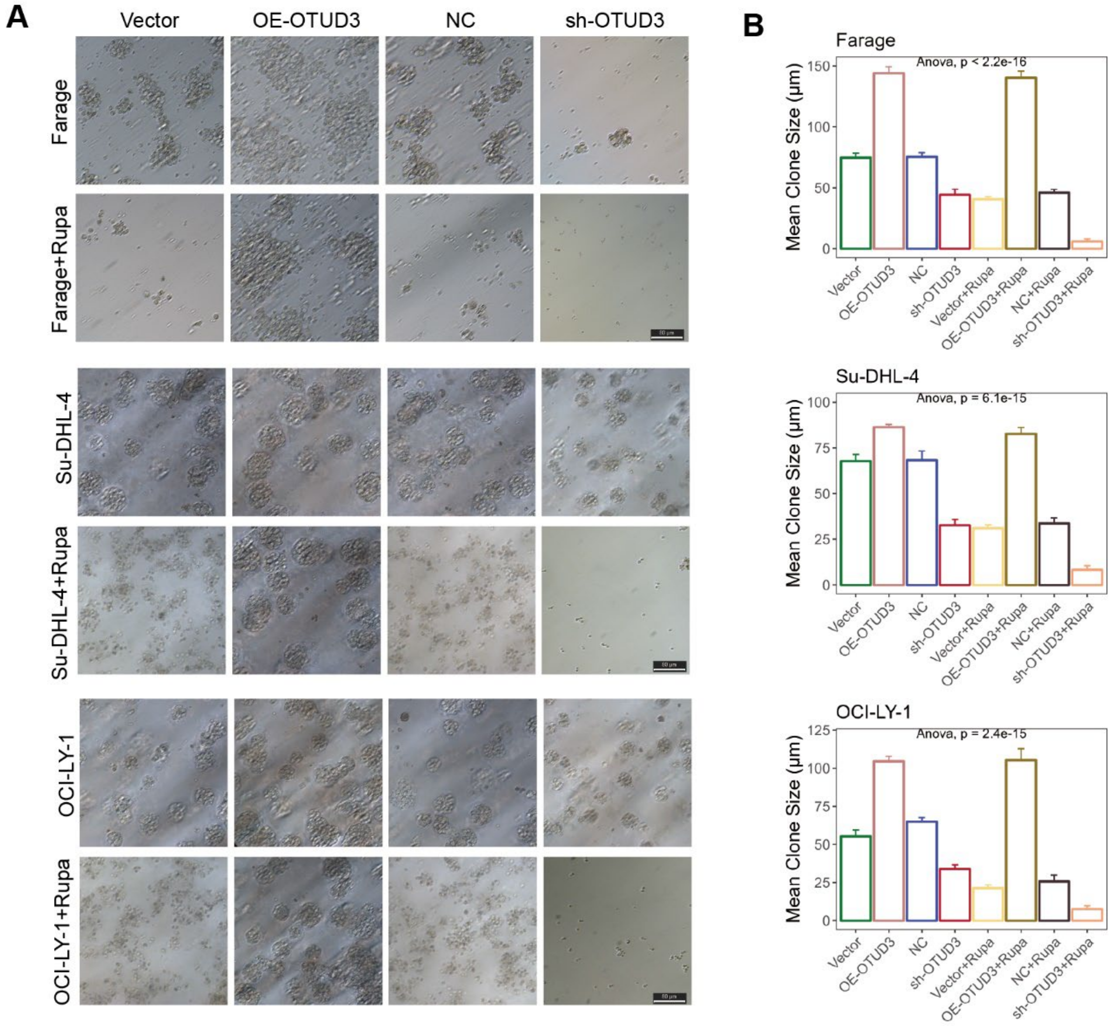

Fig.S8 A, B Clone Assay of all groups.
