# Supplementary material for: Rupatadine-inhibited OTUD3 promotes DLBCL progression and immune evasion through deubiquitinating MYL12A and PD-L1
Source: Cell Death Dis. 2024 Aug 3;15(8):561. doi: 10.1038/s41419-024-06941-x (PMC11297949; doi:10.1038/s41419-024-06941-x)

Figure1B

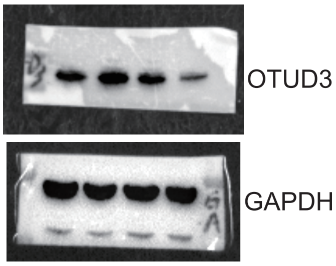

Figure1N

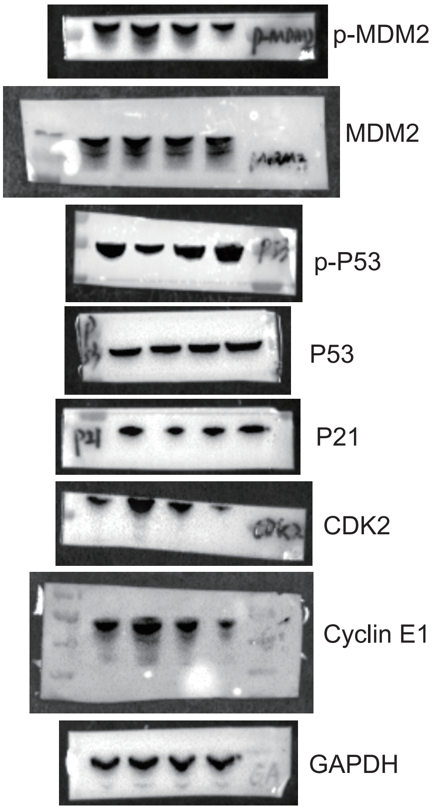

Figure2B

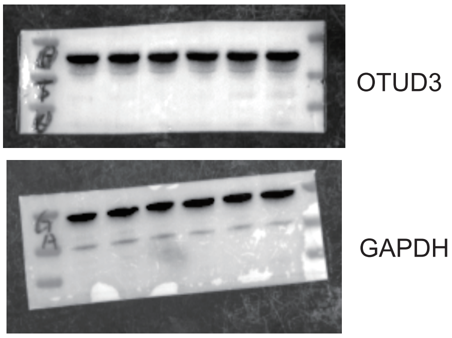

Figure1K

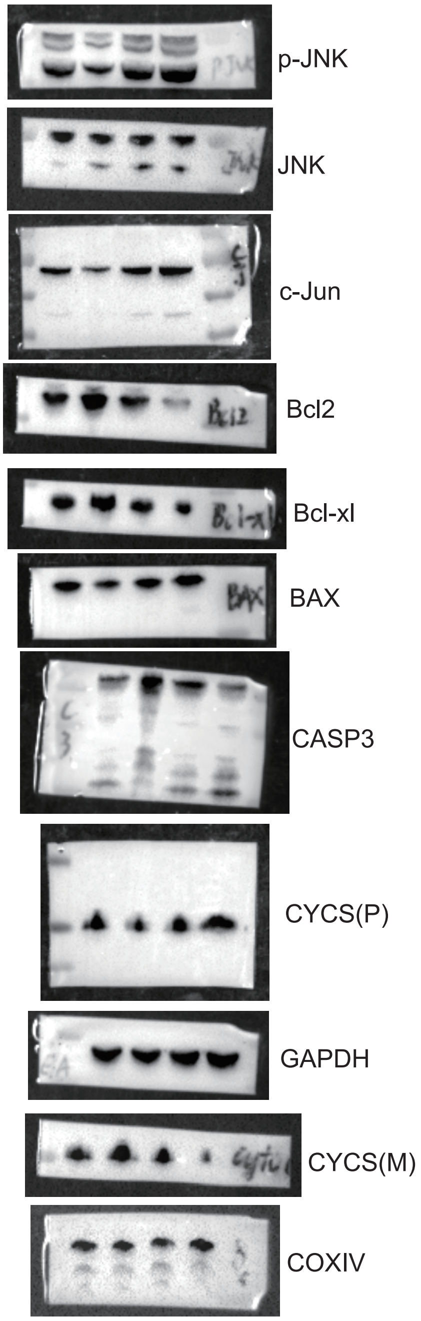

Figure2N

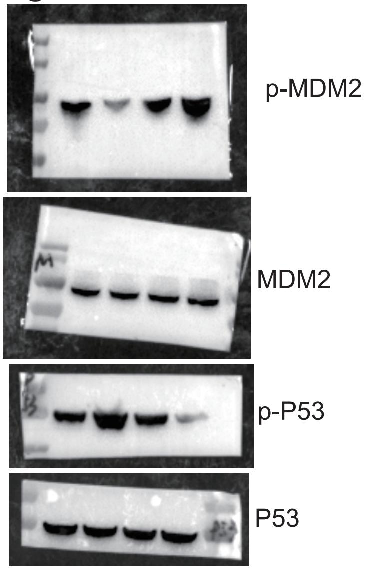

Figure2K

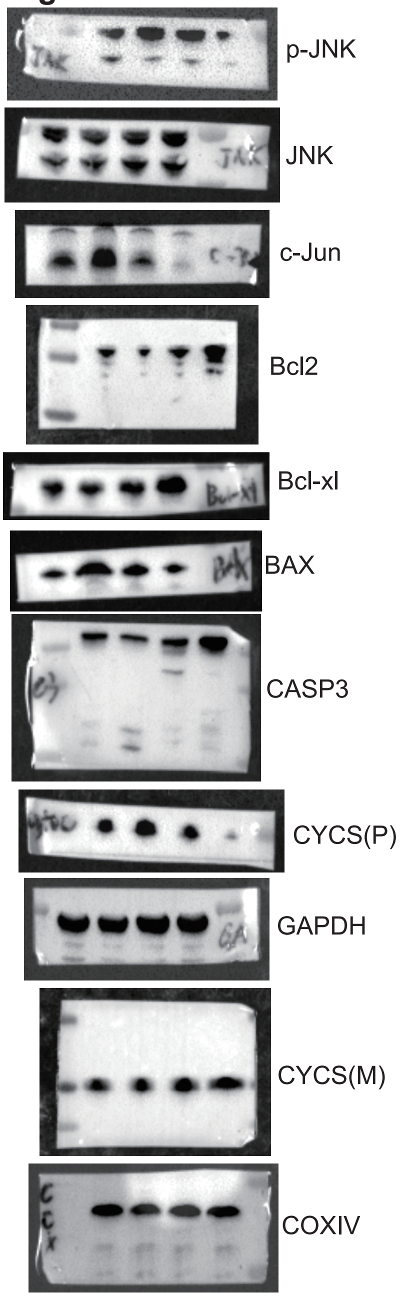

Figure2N

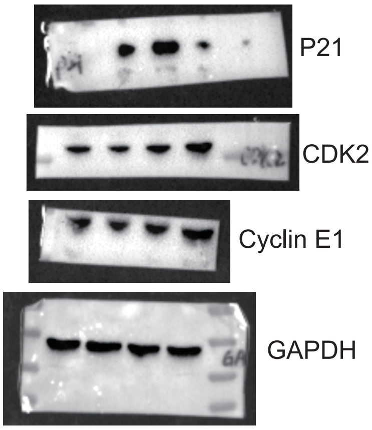

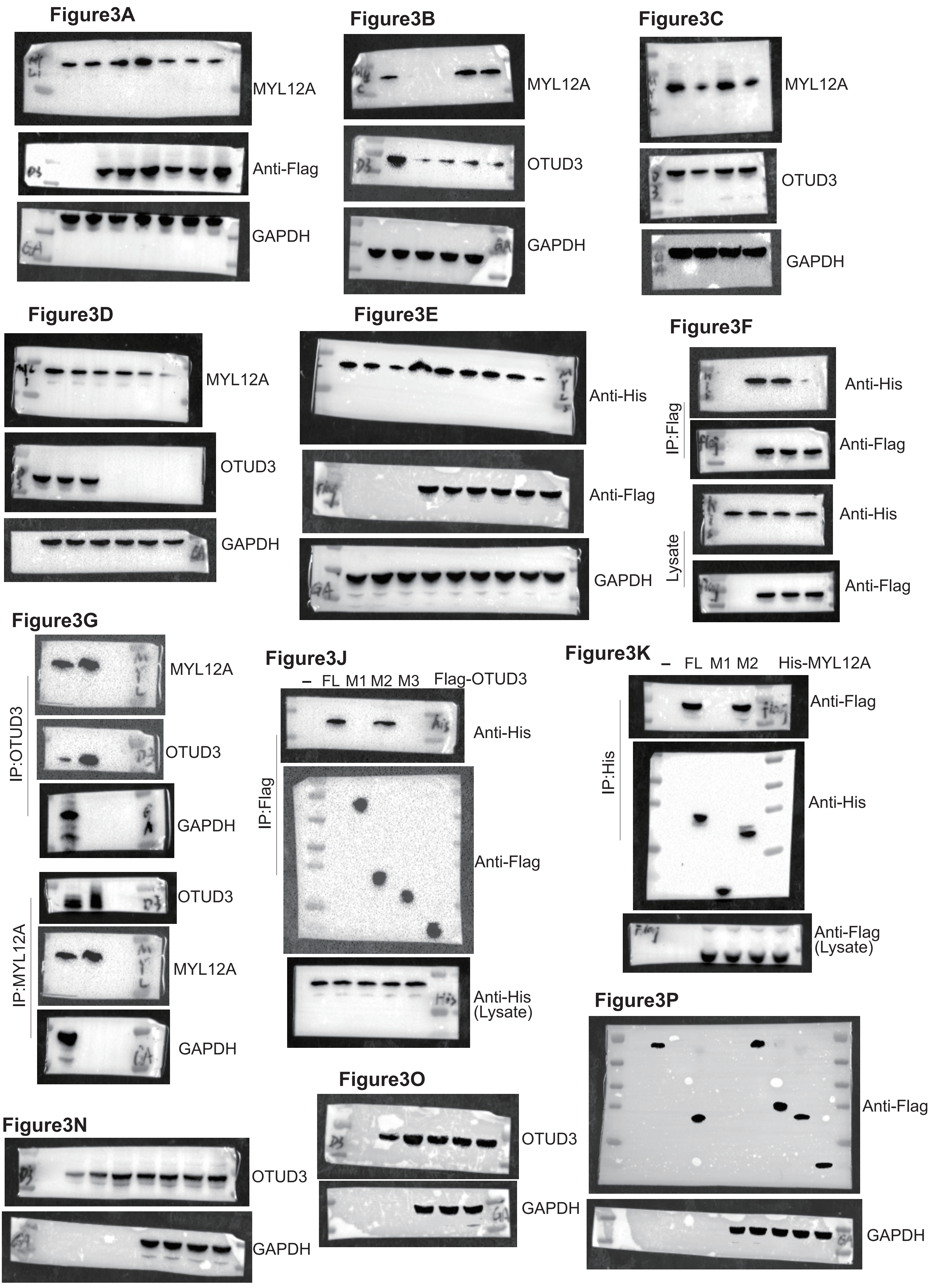

Figure3A
MYL12A
Anti-Flag
GAPDH
Figure3B
MYL12A
OTUD3
GAPDH
Figure3C
MYL12A
OTUD3
GAPDH
Figure3D
MYL12A
OTUD3
GAPDH
Figure3E
Anti-His
Anti-Flag
GAPDH
Figure3F
IP:Flag
Anti-His
Anti-Flag
Lysate
Anti-His
Anti-Flag
Figure3G
IP:OTUD3
MYL12A
OTUD3
GAPDH
IP:MYL12A
OTUD3
MYL12A
GAPDH
Figure3J
– FL M1 M2 M3 Flag-OTUD3
IP:Flag
Anti-His
Anti-Flag
Anti-His (Lysate)
Figure3K
– FL M1 M2 His-MYL12A
IP:His
Anti-Flag
Anti-His
Anti-Flag (Lysate)
Figure3N
OTUD3
GAPDH
Figure3O
OTUD3
GAPDH
Figure3P
Anti-Flag
GAPDH

Figure4A

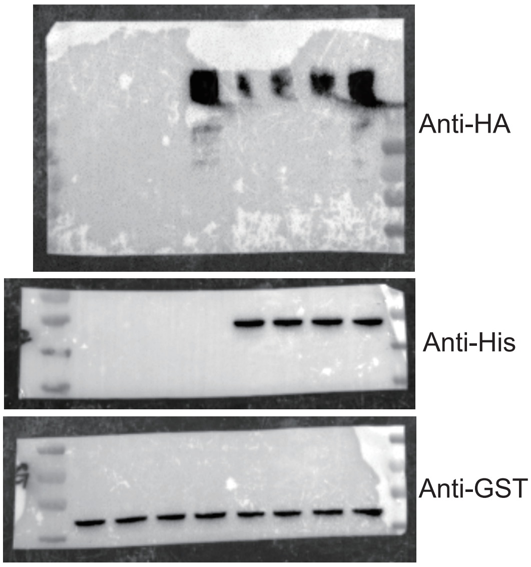

Figure4B

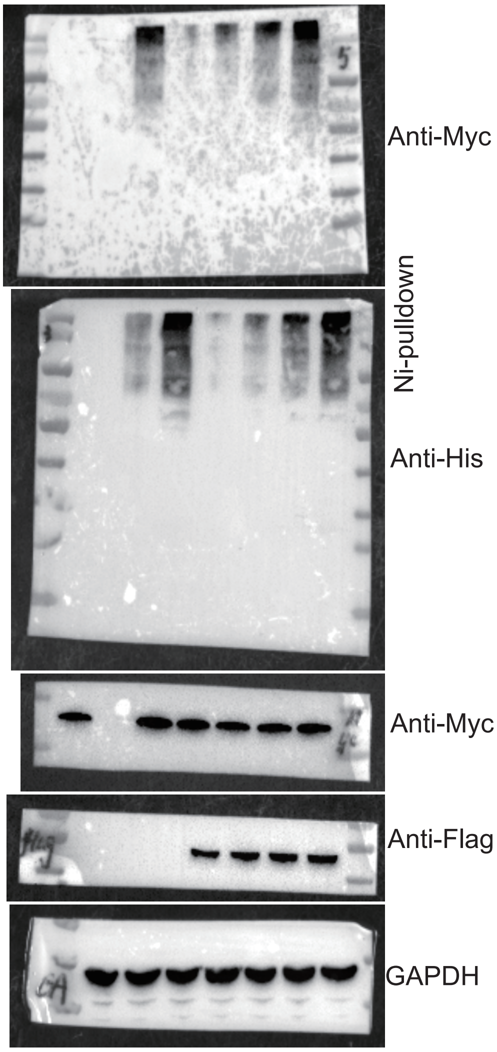

Figure4C

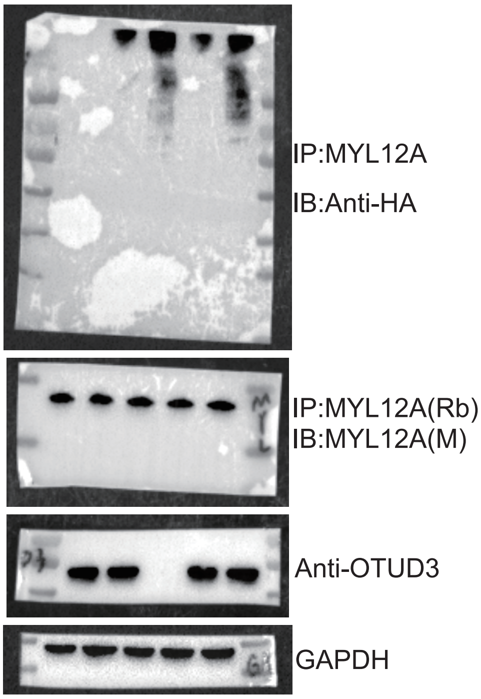

Figure4D

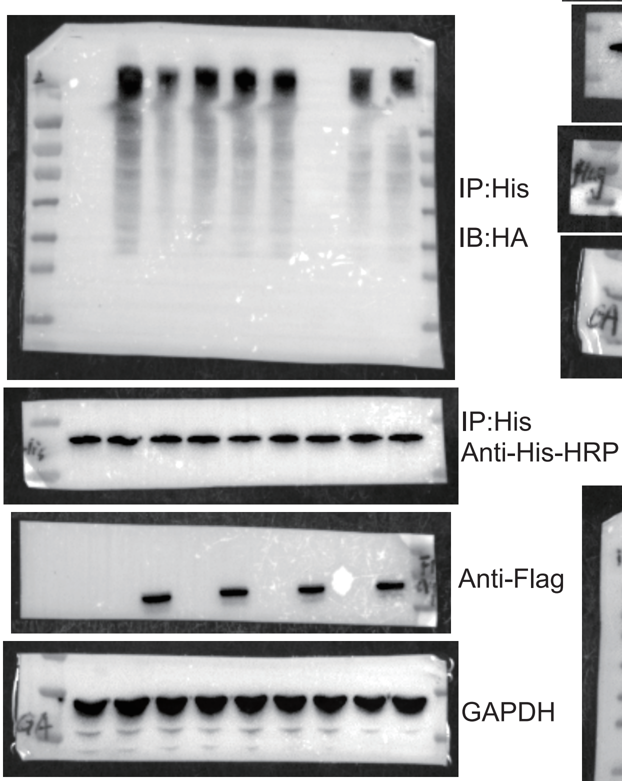

Figure4E

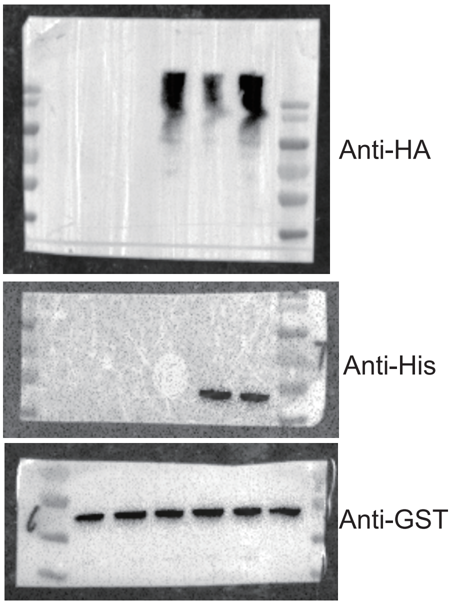

Figure4F

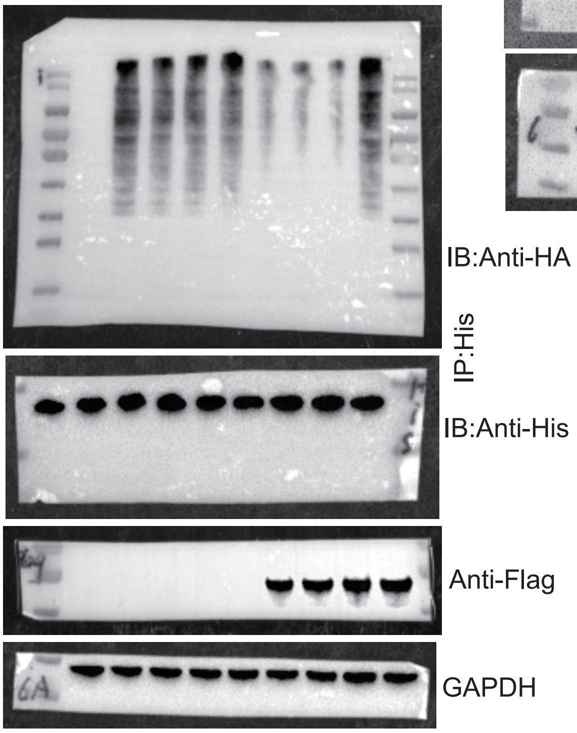

Figure4G

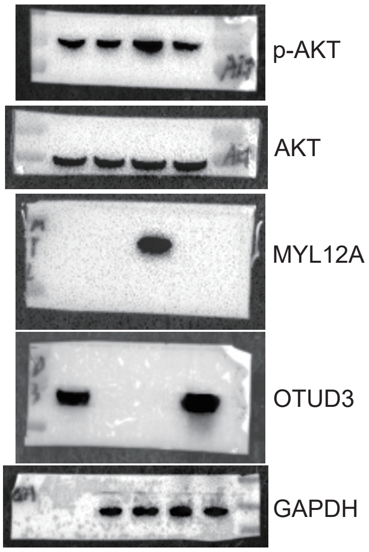

Figure4H

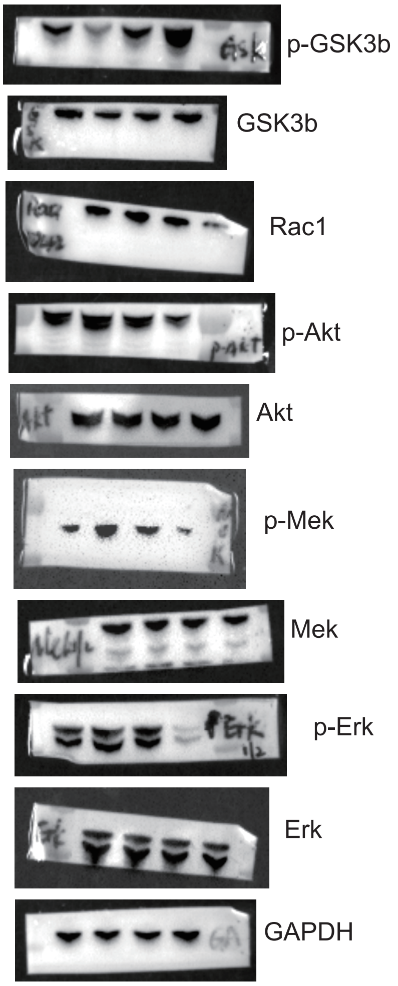

Figure5G

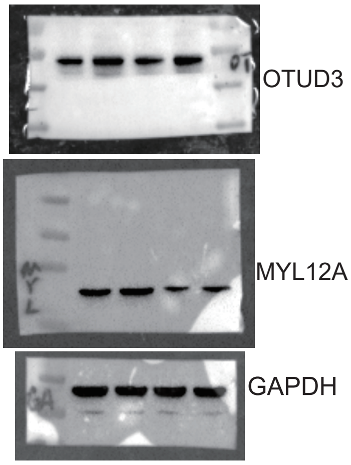

Figure6F

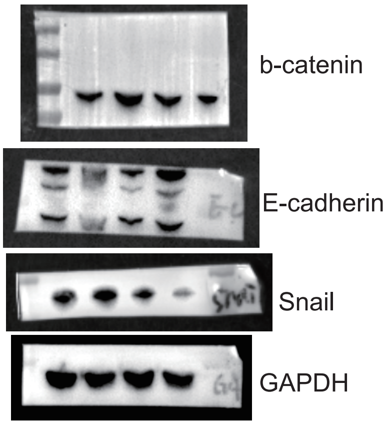

Figure4I

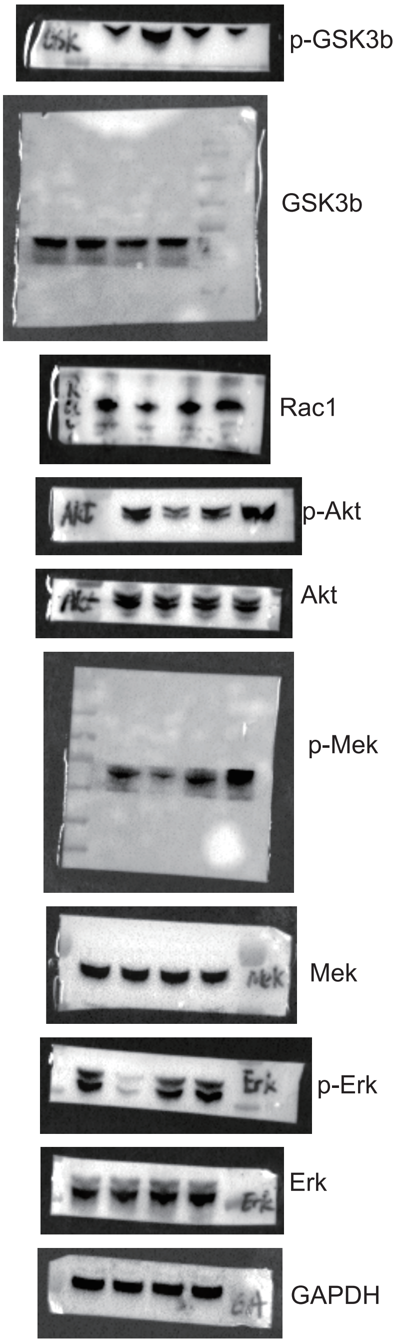

Figure6F

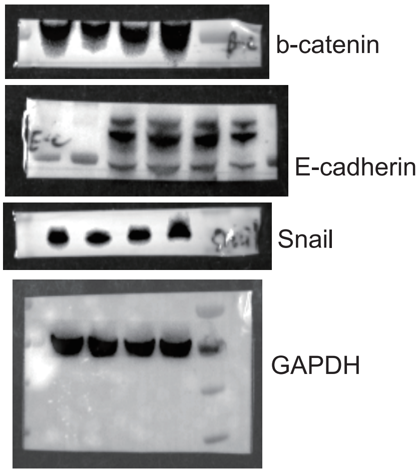

Figure5O

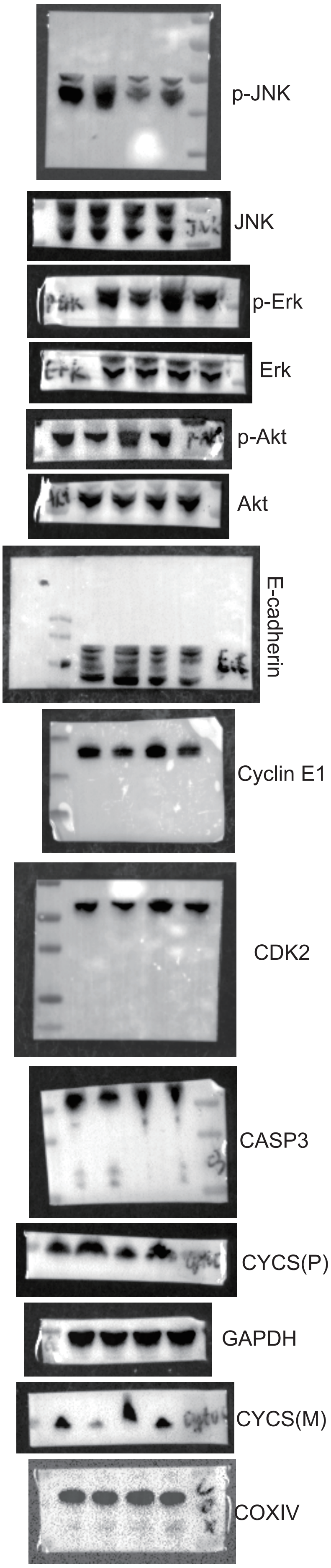

Figure6I

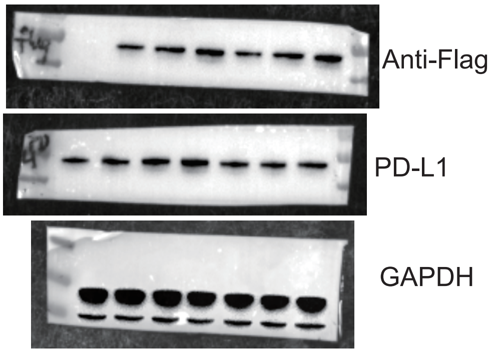

Figure6J

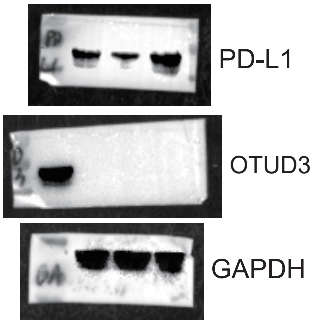

Figure6R

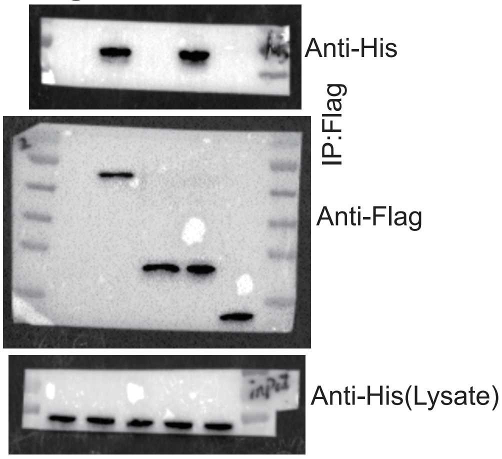

Figure6O

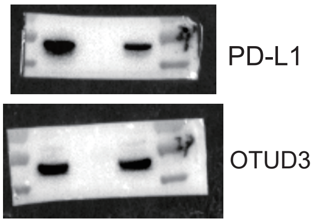

Figure6Q

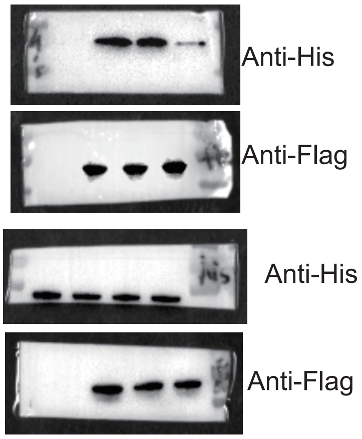

Figure6K

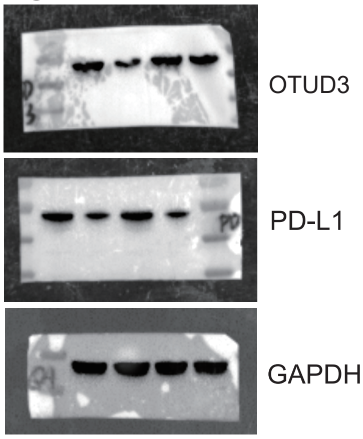

Figure6P

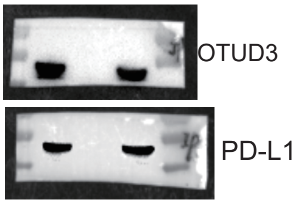

Figure6L

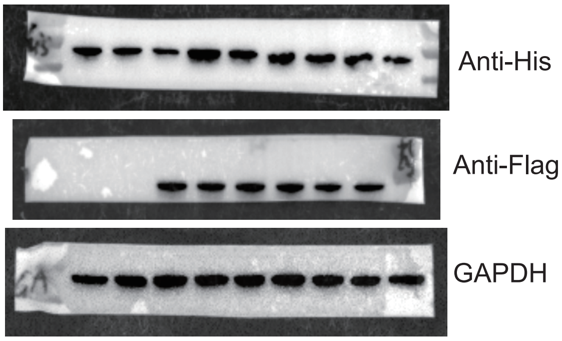

Figure7A

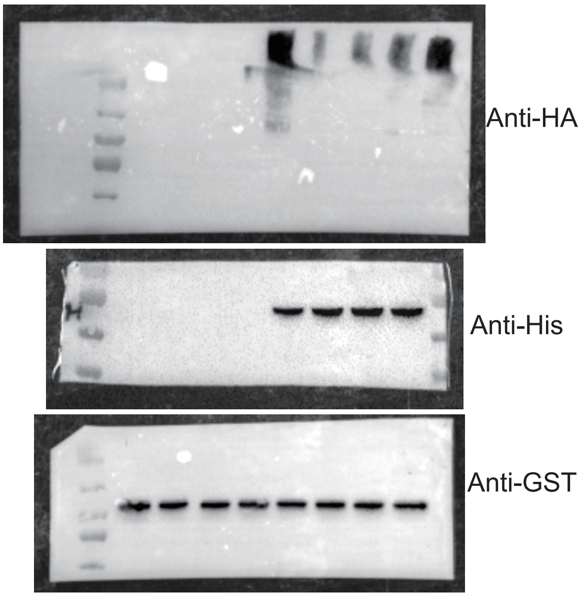

Figure6M

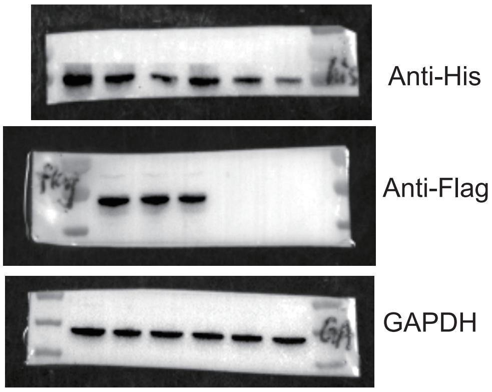

Figure7B

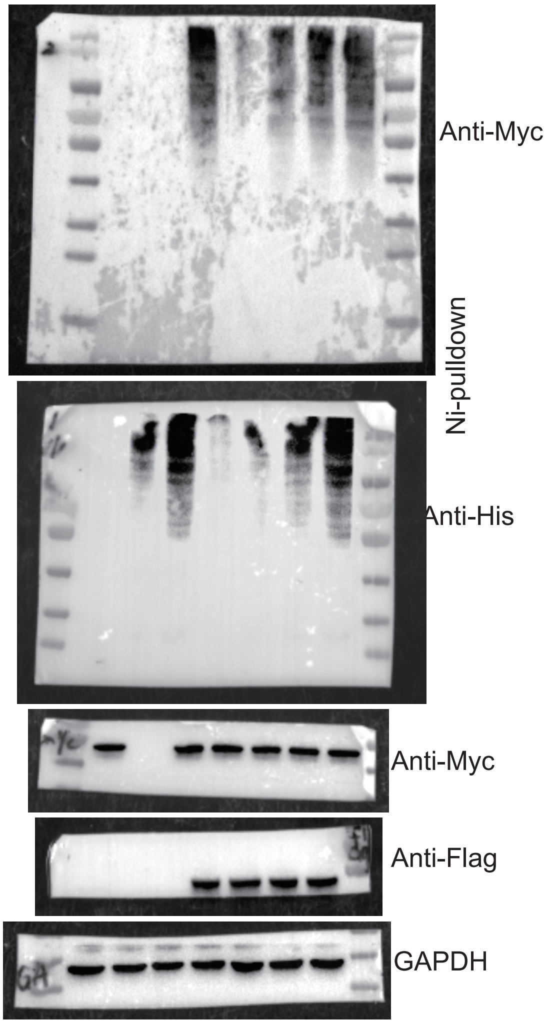

Figure7D

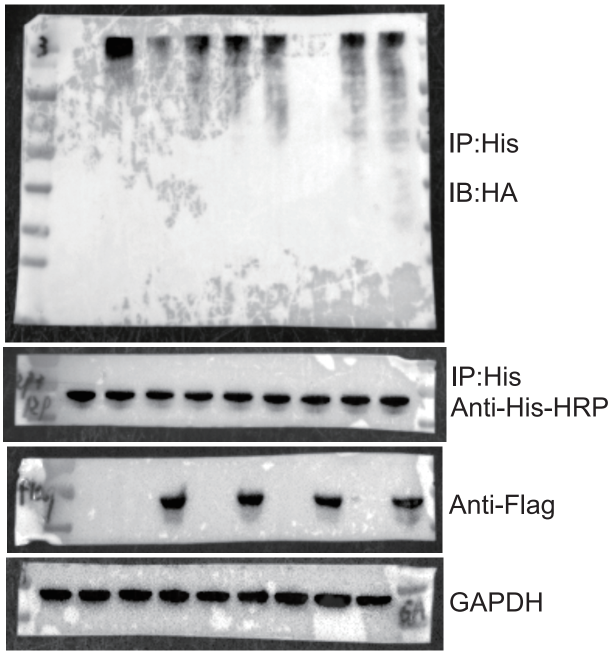

Figure7C

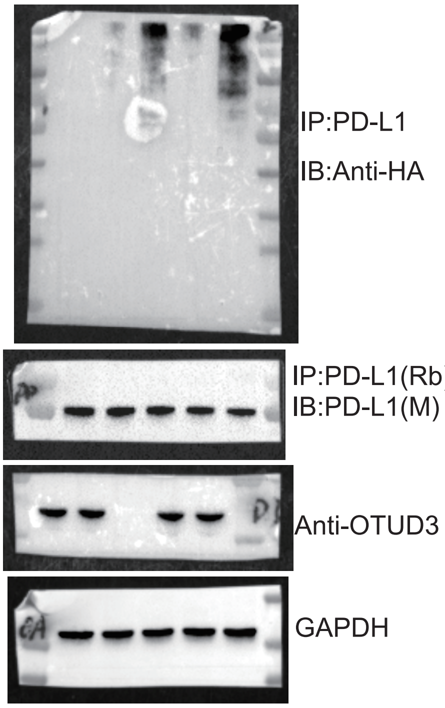

Figure8B

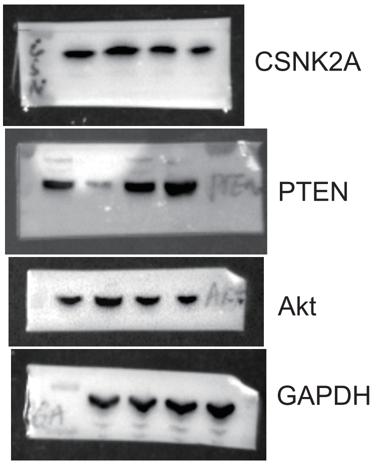

FigureS1D

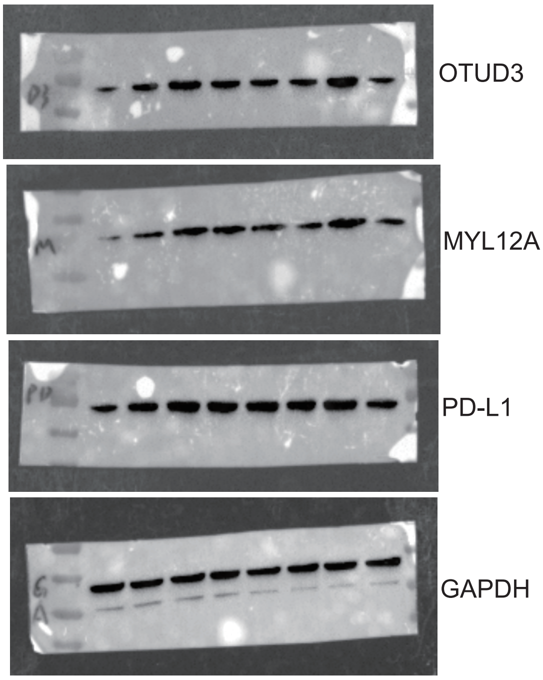

Figure7E

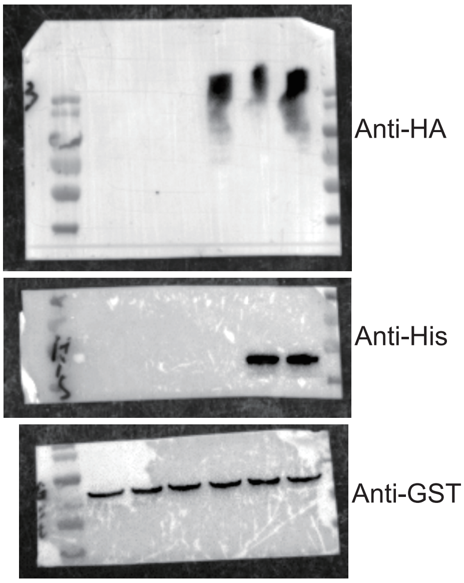

FigureS1F

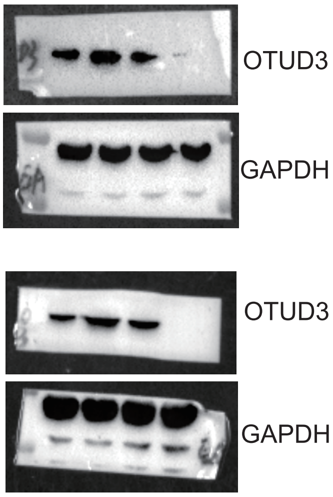

FigureS2G

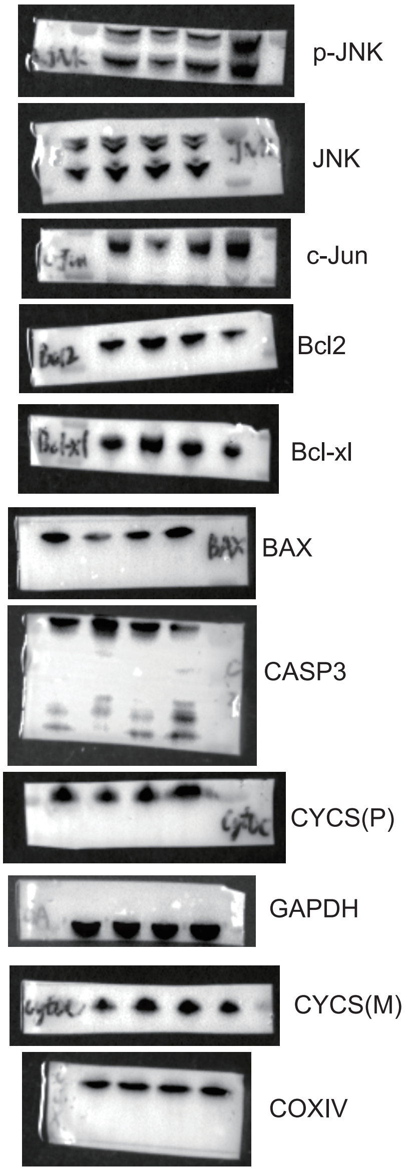

FigureS2H

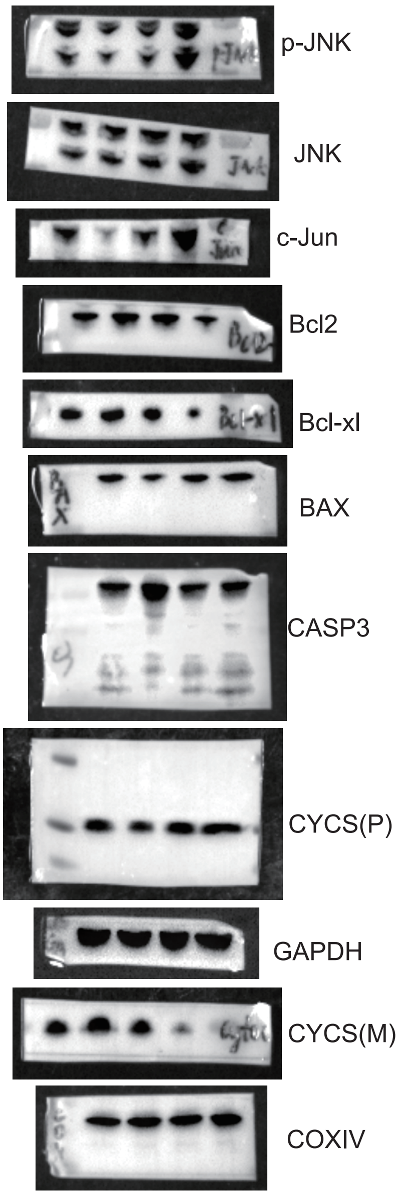

FigureS3A

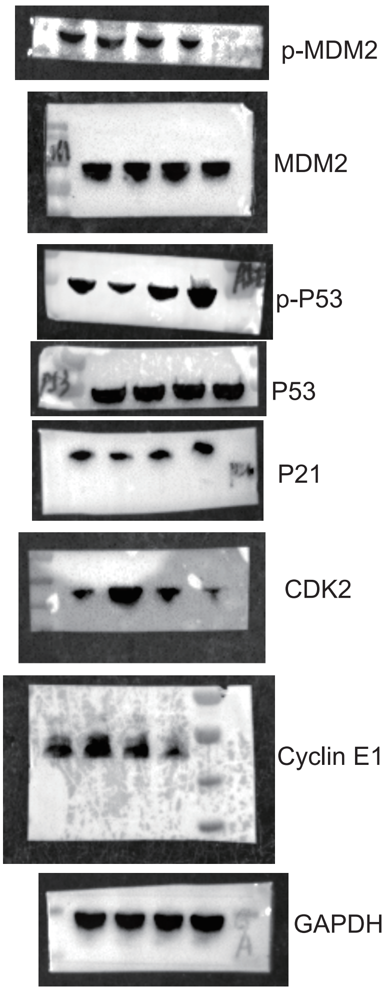

FigureS3A

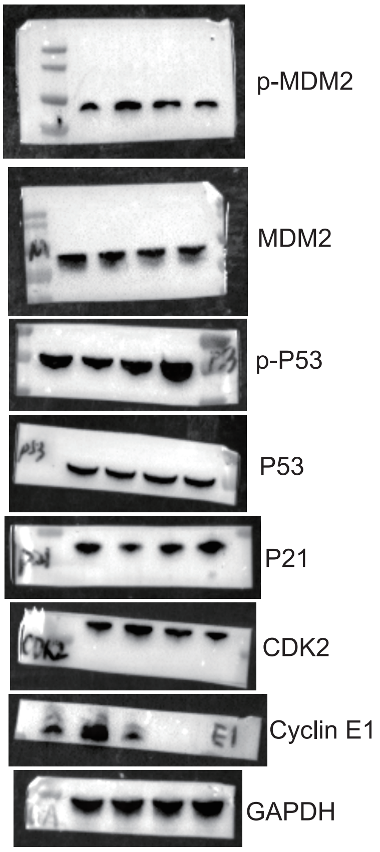

FigureS3B

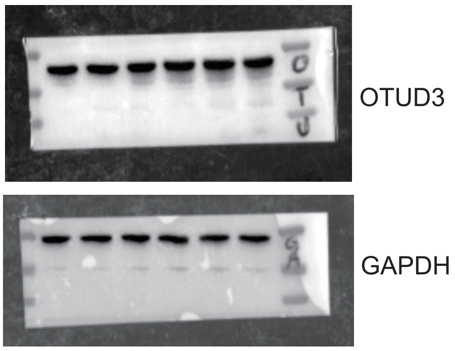

FigureS4I

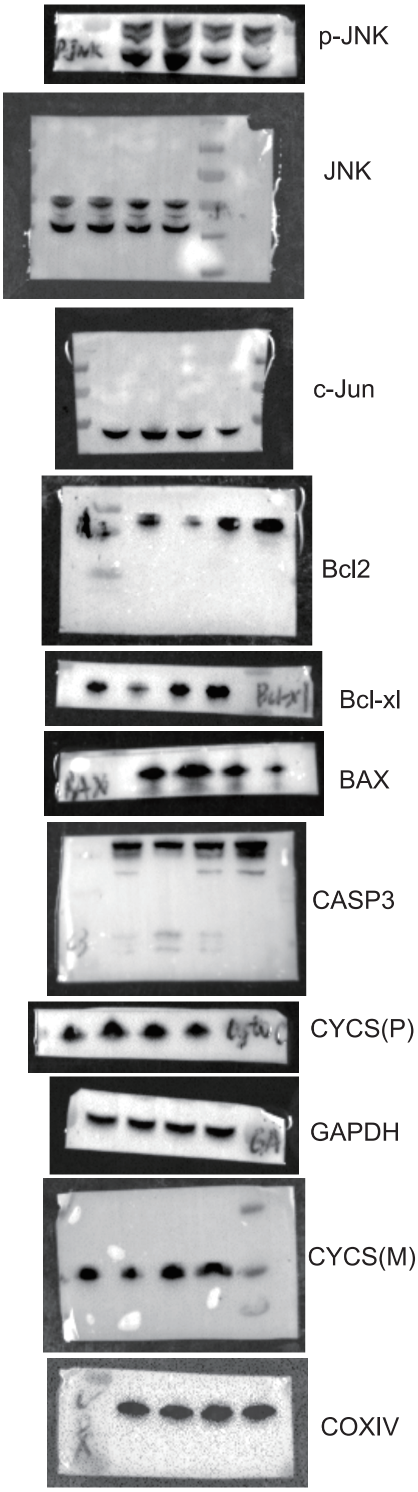

FigureS4J

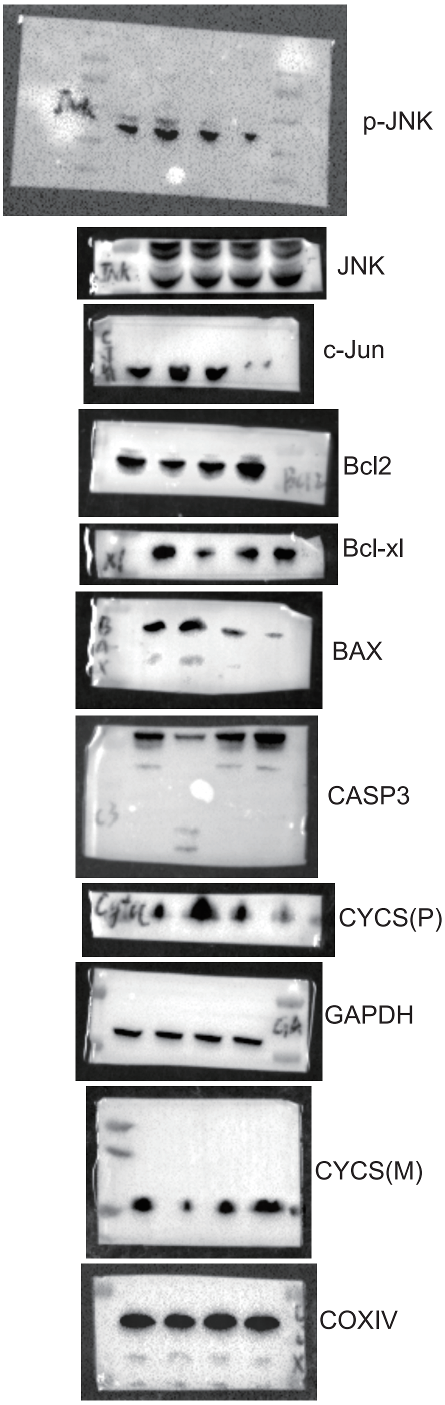

FigureS4N

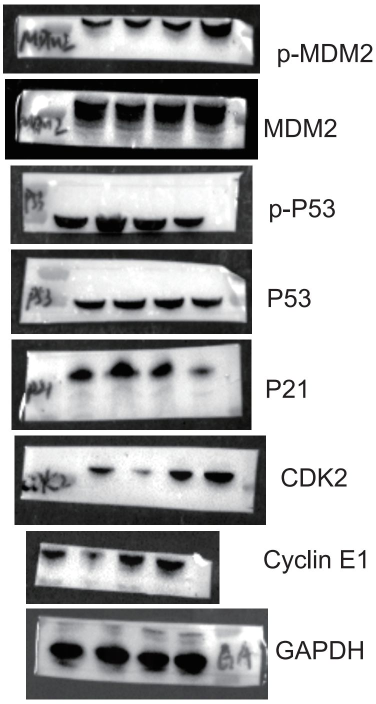

FigureS4O

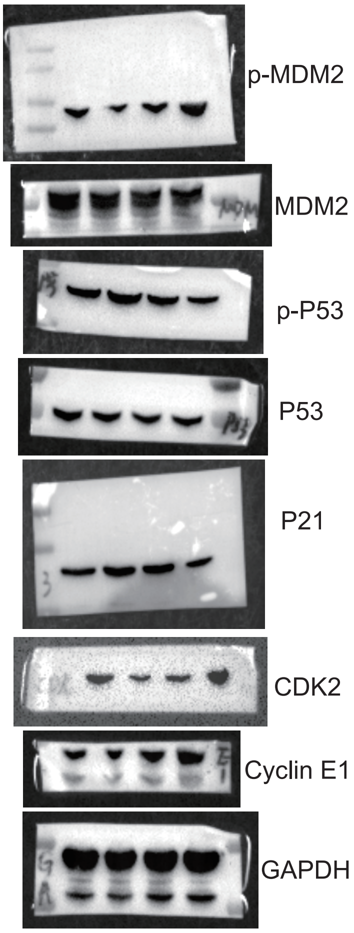

FigureS5A

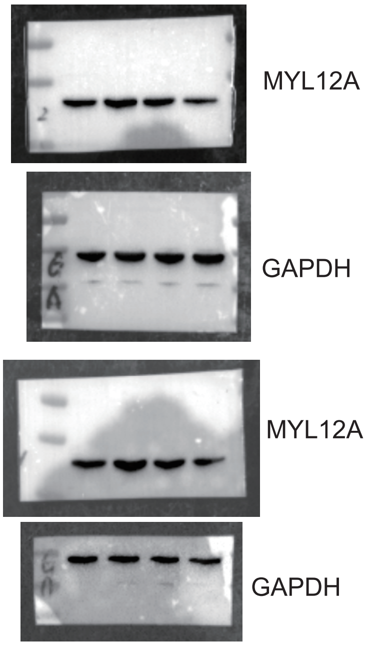

FigureS5H

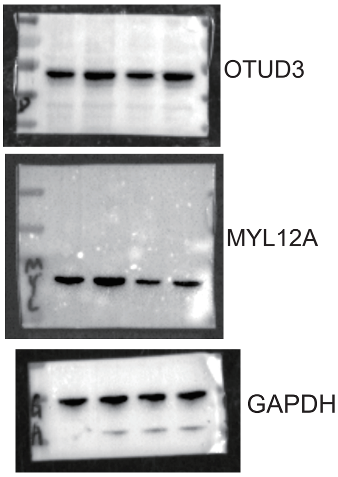

FigureS5N

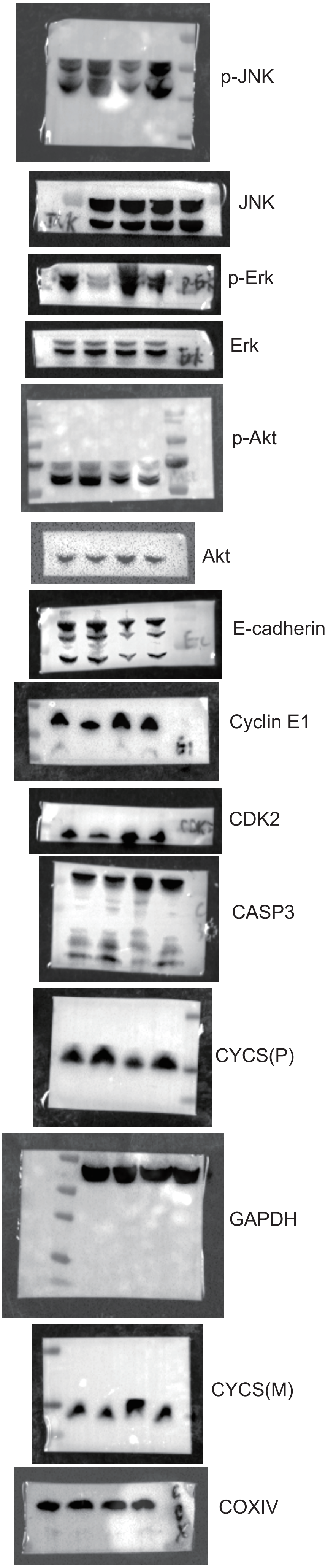

FigureS5O

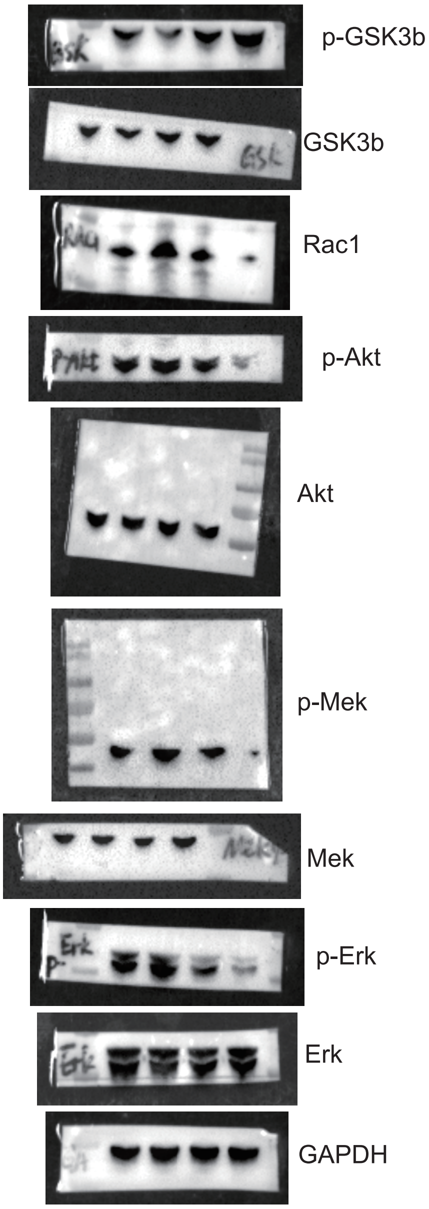

FigureS5O

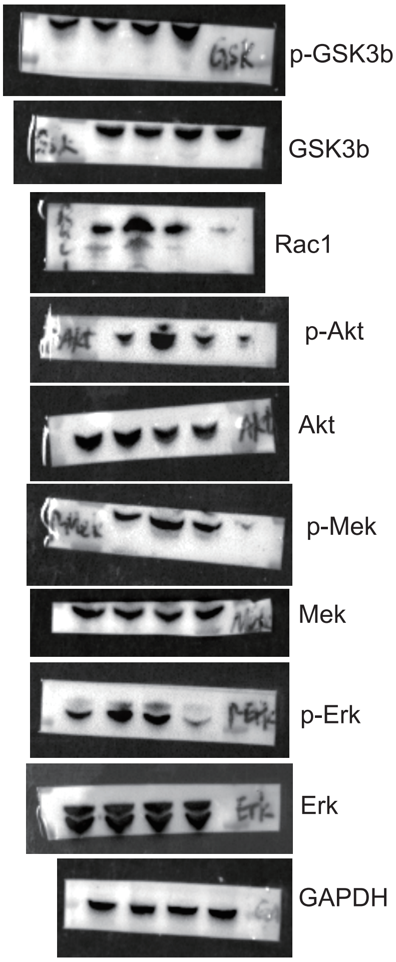

FigureS6A

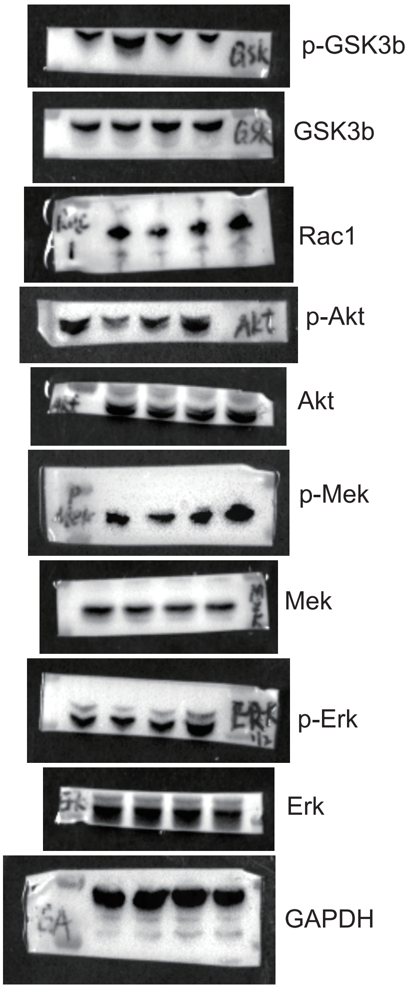

FigureS6A

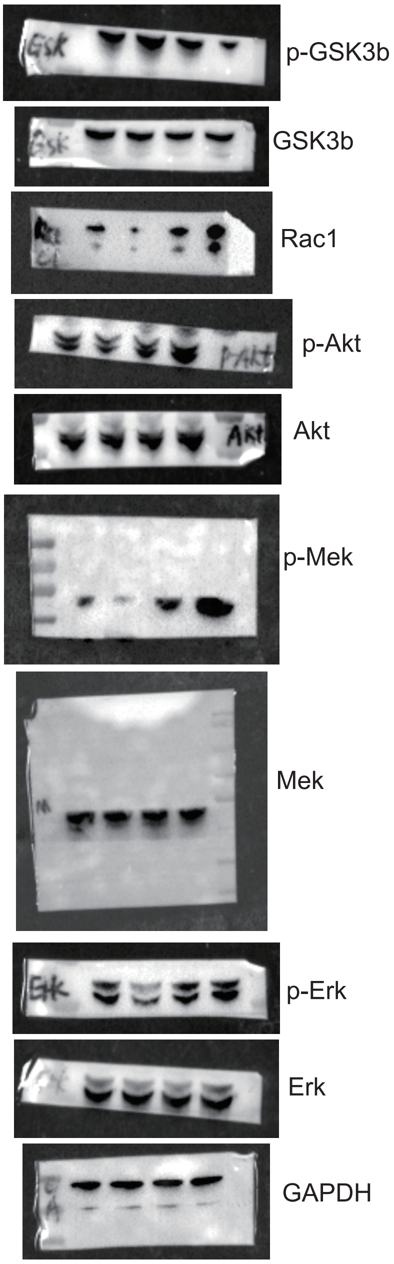

FigureS6F

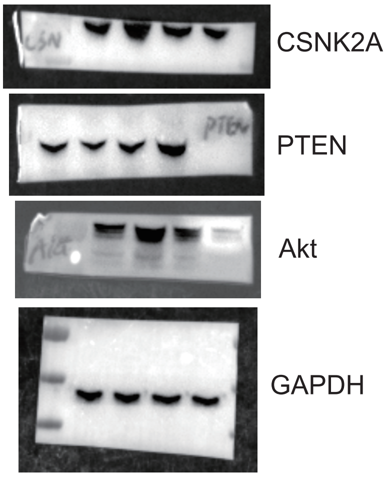

FigureS7C

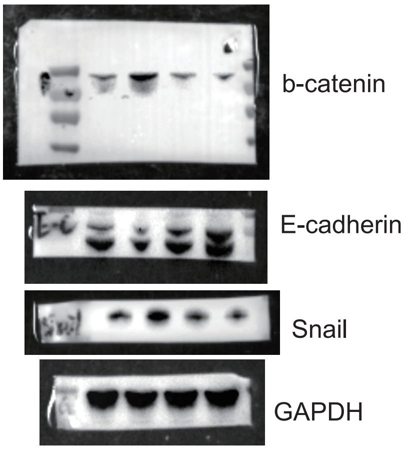

FigureS7E

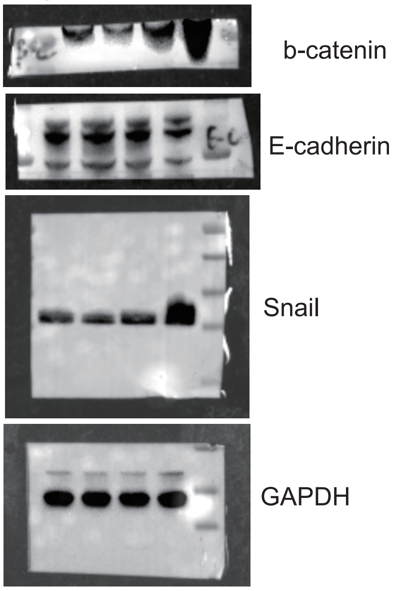

FigureS7D

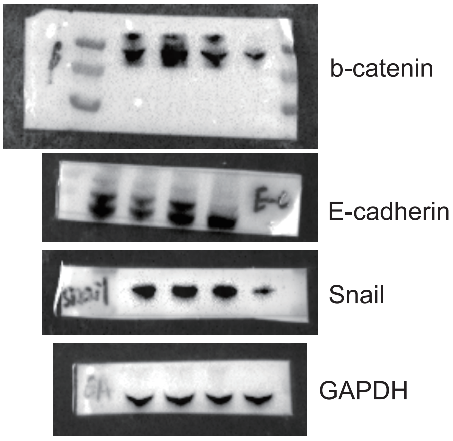

FigureS7F

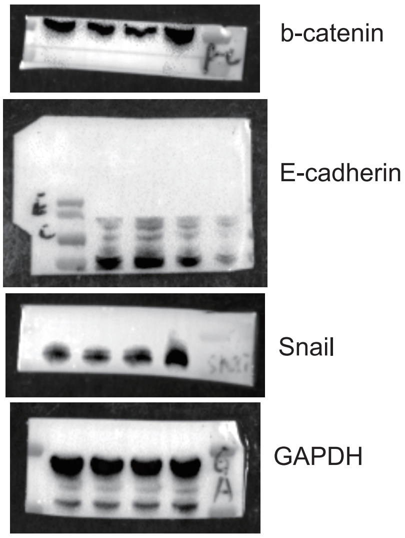

Supplement: Supplementary file 2 — Full and uncropped western blots [file 41419_2024_6941_MOESM2_ESM.pdf]
